# Combination of ginsenoside Rb1 and Rd protects the retina against bright light-induced degeneration

**DOI:** 10.1038/s41598-017-06471-x

**Published:** 2017-07-20

**Authors:** Minjuan Bian, Xiaoye Du, Peiwei Wang, Jingang Cui, Jing Xu, Jiangping Gu, Teng Zhang, Yu Chen

**Affiliations:** 1 Yueyang Hospital and Clinical Research Institute of Integrative Medicine, Shanghai University of Traditional Chinese Medicine, Shanghai, 200437 China; 2 Department of Pharmacy, East China University of Science and Technology, Shanghai, 201203 China

## Abstract

Photoreceptor degeneration is a central pathology of various retinal degenerative diseases which currently lack effective therapies. Antioxidant and anti-inflammatory activities are noted for Panax notoginsenoside saponins (PNS) and related saponin compound(s). However, the photoreceptor protective potentials of PNS or related saponin compound(s) remain unknown. The current study revealed that PNS protected against photoreceptor loss in bright light-exposed BALB/c mice. Combination of ginsenoside Rb1 and Rd, two major saponin compounds of PNS, recapitulated the retinal protection of PNS and attenuated retinal oxidative stress and inflammatory changes. Rb1 or Rd partially alleviated all-*trans*-Retinal-induced oxidative stress in ARPE19 cells. Rb1 or Rd suppressed lipopolysaccharides (LPS)-induced proinflammatory gene expression in ARPE19 and RAW264.7 cells. Rb1 or Rd also modulated the expression of proinflammatory microRNA, miR-155 and its direct target, anti-inflammatory SHIP1, in LPS-stimulated RAW264.7 cells. The retinal expression of miR-155 and SHIP1 was altered preceding extensive retinal damage, which was maintained at normal level by Rb1 and Rd combination. This work shows for the first time that altered expression of miR-155 and SHIP1 are involved in photoreceptor degeneration. Most importantly, novel retinal protective activities of combination of Rb1 and Rd justify further evaluation for the treatment of related retinal degenerative disorders.

## Introduction

Photoreceptor degeneration is causally associated with vision impairment and even blindness. It is a central pathology seen in various retinal degenerative disorders including age-related macular degeneration (AMD), Stargardt disease, a juvenile form of macular degeneration, and retinitis pigmentosa^1, 2^. Effective therapies preventing or ameliorating the progressive loss of photoreceptors remain to be developed. Oxidative stress is one of the essential mechanisms that directly contribute to photoreceptor death^3–5^. In addition, exaggerated inflammatory responses aggravate photoreceptor loss and are critically implicated in the progression of retinal degeneration^6, 7^. Pharmacological agents with antioxidant and anti-inflammatory activities thus hold promises for optimal photoreceptor protection.

MicroRNAs (miRNAs) are small non-coding RNA molecules with gene expression regulatory functions. A miRNA is usually in the approximate length of 21–25 nucleotides and binds the 3′-untranslated region of target RNA transcripts, resulting in mRNA degradation or translational repression, regulating the expression of target genes in a negative manner^8^. MiRNAs play significant roles in nearly all pathophysiological processes^9^. Better understanding of the implications of miRNAs in disease processes may shed new light on the disease mechanisms, which can benefit the mechanism-based therapeutic development.

Panax notoginseng possesses multiple pharmacological activities including antioxidant activity and has a long history of extensive application in the clinical treatment of a wide range of diseases in China^10, 11^. Panax notoginsenoside saponins (PNS), consisting of over 30 types of saponin compounds, form a major class of chemical constituents of Panax notoginseng and contribute significantly to the diverse pharmacological activities of Panax notoginseng^12^. Ginsenoside Rb1 (Rb1), ginsenoside Rg1 (Rg1), ginsenoside Rd (Rd) and notoginsenoside R1 (R1) together represent over 75% of saponins found in PNS. These saponin compounds are naturally occurring antioxidant and are equipped with anti-inflammatory activities. They have been proved to be therapeutically effective in various experimental conditions including neurodegenerative disorders^13–18^. Our previous studies have demonstrated that miRNA-mediated gene expression regulation is associated with the cardioprotective, anti-atherosclerotic and anti-tumor activities of PNS, R1 and Rd^19–22^. However, the pharmacological implications of PNS and related saponin compound(s) in photoreceptor degeneration remain to be examined. The current study thus investigated the retinal protective activity of PNS and underlying mechanisms of PNS-related saponin compound(s) in a mouse model characterized by bright light-induced photoreceptor degeneration.

## Results

### PNS treatment prevented the development of bright light-induced photoreceptor degeneration in mouse

**Figure 1:**
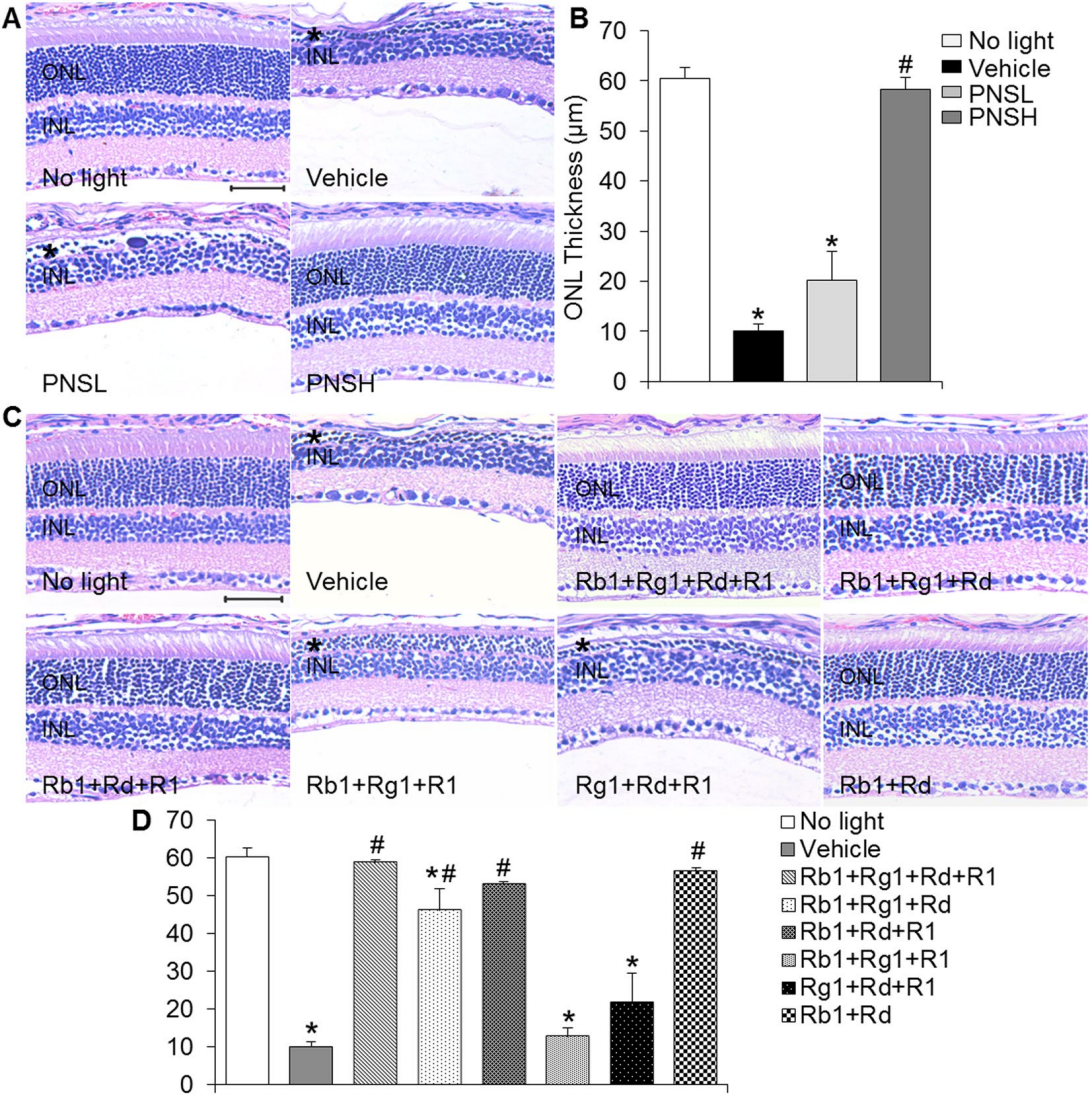
PNS or natural combination of major saponin components of PNS protected retinas from developing bright light-induced degeneration. (**A**) Dar-adapted BALB/c mice were pretreated with saline vehicle (Vehicle), PNS at 50 mg/kg bw (PNSL) and 200 mg/kg bw (PNSH) 30 min before bright light exposure. Saline-treated mice unexposed to bright light (No light) were included as normal controls. Paraffin sections made from enucleated eyes were subject to histological examination by H&E staining (n = 4 per group). (**B**) The ONL thickness was measured at 500 μm from optic nerve head (ONH) in the superior retina. (**C**) Dark-adapted BALB/c mice were pretreated with the indicated combinations of the major saponins in PNS, which included Rb1 + Rg1 + Rd + R1, Rb1 + Rg1 + Rd, Rb1 + Rd + R1, Rb1 + Rg1 + R1, Rg1 + Rd + R1 and Rb1 + Rd. The dose of each saponin in the combinatorial treatment was determined according to its respective content in PNS and the effective dose of PNS at protecting against bright light-induced photoreceptor degeneration, which was indicated as following: Rb1, 65 mg/kg bw, Rg1, 50 mg/kg bw, Rd, 22.5 mg/kg bw and R1, 18 mg/kg bw. H&E staining was performed to examine retinal morphology (n = 4–8 per group). (**D**) The ONL thickness was measured at 500 μm from optic nerve head (ONH) in the superior retina. Asterisk indicated ONL manifesting bright light-induced degeneration. INL, inner nuclear layer; ONH, optic nerve head; ONL, outer nuclear layer. Scale bar: 50 μm. The data were expressed as the mean ± S.E.M (n = 3–8 per group). *Compared to that from No light, *p* < 0.05; ^#^compared to that from Vehicle, *p* < 0.05.

The retinal protective activity of PNS was examined in a mouse model of bright light-induced retinal degeneration. Dark-adapted BALB/c mice were exposed to bright light at the intensity of 10,000 lux for 30 min. PNS was intraperitoneally administered 30 min before bright light exposure at the dose of 50 and 200 mg/kg bw. OCT imaging was performed 7 d after bright light exposure to assess the changes in retinal structure. As shown in Supplementary Fig. S1, compared to that from vehicle-treated mice unexposed to bright light, outer nuclear layer (ONL) was severely impaired in bright light-exposed vehicle-treated mice. However, well-preserved ONL structure was readily observed in bright light-exposed mice treated with PNS at 200 mg/kg bw. No such protection was observed in bright light-exposed mice treated with PNS at 50 mg/kg bw. Histological examination was then performed to better visualize the gross morphology of the retina, followed by measurement of ONL thickness to quantify the changes in photoreceptors. As shown in Fig. 1A,B, compared to that from vehicle-treated mice unexposed to bright light, overtly disrupted photoreceptor morphology was observed in bright light-exposed vehicle-treated mice, which was characterized by diminished ONL, outer segment (OS) and inner segment (IS). No photoreceptor protection was observed in bright light-exposed mice treated with PNS at 50 mg/kg bw. However, remarkable preservation of photoreceptor morphology was observed in bright light-exposed mice treated with PNS at 200 mg/kg bw, which was evidenced by well-preserved ONL, OS and IS. These results indicated that PNS treatment was effective at protecting photoreceptors from bright light-induced degeneration in mouse.

### Combination of Rb1 and Rd protected photoreceptors from developing bright light-induced degeneration in mouse

To better understand the chemical mechanism underlying the retinal protective activity of PNS, retinal protective effect of the major saponin compounds of PNS, namely Rb1, Rg1, Rd and R1^23^, was further examined individually in bright light-exposed mice. The dose of individual saponin compound, 65, 50, 22.5 and 25 mg/kg bw for Rb1, Rg1, Rd and R1, respectively, was determined mainly based on the respective content in PNS used for the current study^23^ and the effective dose of PNS at protecting photoreceptors against bright light-induced degeneration (200 mg/kg bw) as shown in Fig. 1A,B. As shown in Supplementary Fig. S1, OCT imaging showed that when administered individually, none of the tested compounds provided retinal protection against bright light-induced degeneration in a similar fashion as that conferred by PNS treatment delivered at 200 mg/kg bw. These results suggested that more than one type of saponin compounds could be required to recapitulate the retinal protective activity of PNS. Therefore, retinal protective effects of combinations consisting of 4 major saponin compounds were further tested in bright light-exposed mice. OCT imaging showed that consistent with that observed from PNS treatment, combination of 4 major saponin compounds, Rg1, Rb1, Rd and R1 resulted in significant protection of photoreceptors against bright light-induced degeneration (Supplementary Fig. S1), which was also evidenced by histological examination (Fig. 1C,D). Moreover, retinal protection was observed in bright light-exposed mice treated with the combination of Rb1, Rg1 and Rd or that of Rb1, Rd and R1 but not with combination of Rb1, Rg1 and R1 or Rg1, Rd and R1 (Supplementary Fig. S1 and Fig. 1C,D). These results led to the hypothesis that both Rb1 and Rd are required for executing the retinal protection conferred by PNS in bright light-exposed mice. The retinal protective effect of combination of Rb1 (65 mg/kg bw) and Rd (22.5 mg/kg bw) (indicated as natural combination in the following text unless otherwise specified) was thus further tested in bright light-exposed mice. OCT imaging revealed nearly complete photoreceptor protection in bright light-exposed mice treated with combination of Rb1 and Rd (Supplementary Fig. S1), which was confirmed by histological examination (Fig. 1C,D). These results collectively indicated that Rb1 and Rd were the major saponin compounds responsible for the photoreceptor protective activity of PNS in bright light-exposed mice.

To further determine which saponin compound in the combination of Rb1 or Rd played a major role in conferring retinal protection, mono treatment of Rb1 and Rd was first administered by doubling the doses used in natural combination, specifically, Rb1 was administered at 130 mg/kg bw (Rb1H) and Rd was delivered at 45 mg/kg bw (RdH). As shown in Supplementary Fig. S1, no significant protection of photoreceptors was observed in bright light-exposed mice Rb1H-treated or RdH-treated mice. Furthermore, varied dosing regimens of Rb1 and Rd combination were tested in bright light-exposed mice. Firstly, Rb1 was administered at a constant dose of 65 mg/kg bw, which was photoreceptor protective when delivered together with Rd at 22.5 mg/kg bw. The dose for Rd was lowered from 22.5 mg/g bw to 15 mg/kg bw (Rb + RdL1) and 7.5 mg/kg bw (Rb1 + RdL2). Secondly, Rd was administered at a constant dose of 22.5 mg/kg bw and Rb1 was administered at lowered dose of 44 mg/kg bw (Rd + Rb1L1) or 22 mg/kg bw (Rd + Rb1L2). As shown in Supplementary Fig. S2A, OCT imaging revealed that decreasing the dose of either Rb1 or Rd in combinatorial treatment modality resulted in similarly partial photoreceptor protection in bright light-exposed mice. These observations were confirmed by histological examinations (Supplementary Fig. S2B,C). These results suggested that both Rb1 and Rd were critical for optimal photoreceptor protection in bright light-exposed mice.

### Natural combination of Rb1 and Rd protected rod and cone photoreceptors from bright light-induced impairment

**Figure 2:**
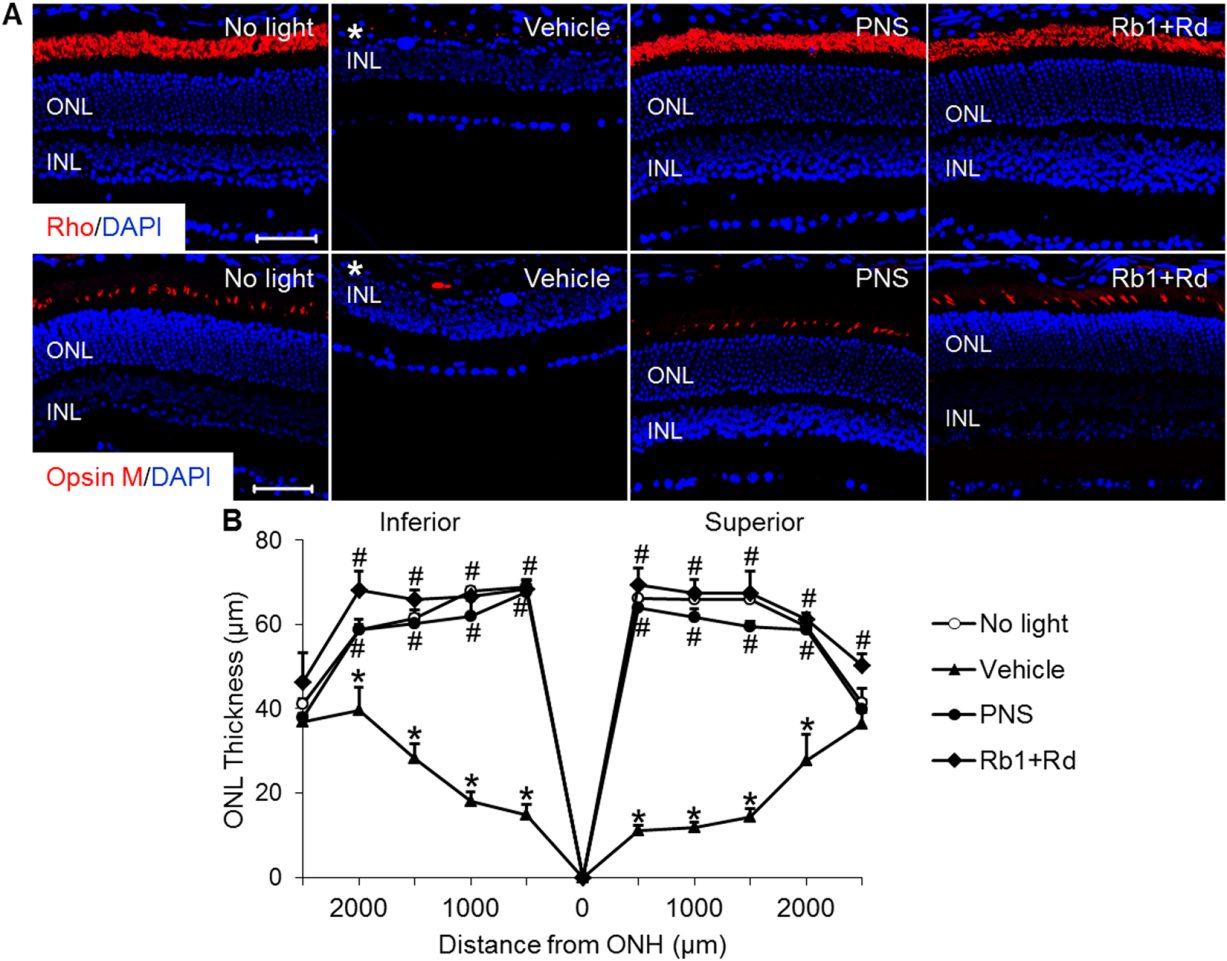
Treatment of PNS or combination of Rb1 and Rd prevented the loss of rods and cones in bright light-exposed mice. Dark-adapted BALB/c mice were pretreated with saline vehicle (Vehicle), PNS at 200 mg/kg bw (PNS) or combination of Rb1 (65 mg/kg bw) and Rd (22.5 mg/kg bw) 30 min before bright light exposure. (**A**) Eyes were enucleated from vehicle-treated mice unexposed to bright light (No light) and the bright light-exposed mice with the indicated treatment 7 d after illumination and paraffin sections were made for IHC assessment of the retinal expression of Rho and opsin M (in red). Counterstaining with DAPI (in blue) was also performed. (**B**) ONL thickness across the retina was measured in DAPI-stained paraffin sections. Asterisk indicated diminished ONL. INL, inner nuclear layer; ONH, optic nerve head; ONL, outer nuclear layer. Scale bar: 50 μm. The data were expressed as the mean ± S.E.M (n = 4–8 per group). *Compared to that from No light, *p* < 0.05; ^#^compared to that from Vehicle, *p* < 0.05.

Immunohistochemical examination of retinal rhodopsin (Rho) and mid-wave length sensitive cone opsin (opsin M) was performed to better characterize the protective effects of natural combination of Rb1 and Rd on rod and cone photoreceptors in bright light-exposed mice. As shown in Fig. 2A, the immunoreactivity of Rho and Opsin M was readily observed in the photoreceptors in vehicle-treated mice unexposed to bright light. The immunoreactivity of Rho or Opsin M, however, was barely detectable in the retinas from bright light-exposed vehicle-treated mice, which validated bright light-induced severe impairment of both rod and cone photoreceptors. In distinct contrast, in bright light-exposed mice treated with natural combination of Rb1 and Rd and those treated with PNS at 200 mg/kg bw, Rho and Opsin M labeling exhibited similar pattern as that observed in vehicle-treated mice unexposed to bright light. Quantification of ONL thickness after DAPI staining revealed remarkable reduction in the thickness of ONL in bright light-exposed vehicle-treated mice compared to that in the mice unexposed to bright light, whereas the ONL thickness was significantly preserved in bright light-exposed mice treated with natural combination of Rb1 and Rd or PNS at 200 mg/kg bw (Fig. 2B). These results confirmed the notion that natural combination of Rb1 and Rd recapitulated the morphological photoreceptor protection conferred by PNS.

**Figure 3:**
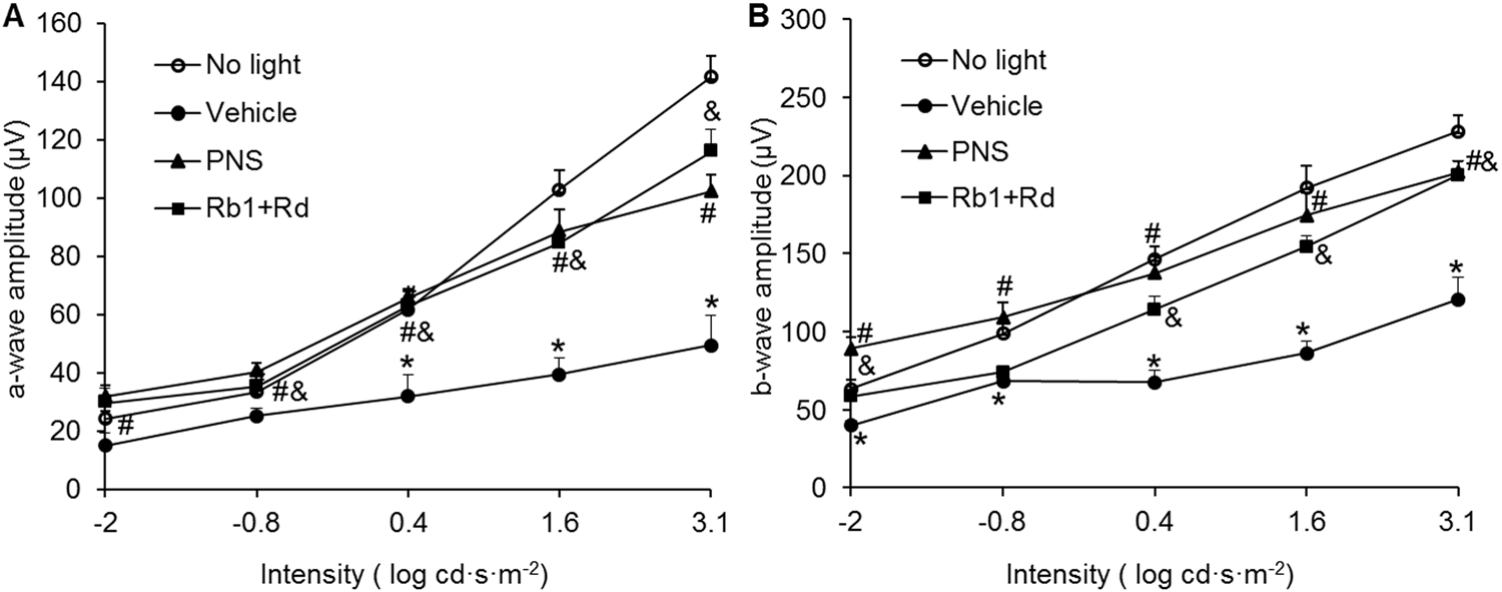
PNS or Rb1 and Rd combination preserved retinal function in bright light-exposed mice. BALB/c mice were treated with vehicle, PNS at 200 mg/kg bw, or natural combination of Rb1 and Rd 30 min before bright light exposure. Retinal function was examined 10 d after bright light exposure. Scotopic ERGs were recorded and amplitudes of a-wave (**A**) and b-wave (**B**) were plotted. Data were expressed as mean ± S.E.M (n = 4–5 per group). *Compared to that from No light, *p* < 0.05; ^#^PNS treatment was compared to that from Vehicle, *p* < 0.05; ^&^Combination treatment of Rb1 and Rd was compared to that from Vehicle, *p* < 0.05.

ERG was further performed to evaluate the retinal function. As shown in Fig. 3, bright light exposure resulted in significant reductions in the scotopic a-wave and b-wave amplitudes in vehicle-treated mice compared to that observed in the mice unexposed to bright light. In contrast, both scotopic a-wave and b-wave amplitudes were well preserved in bright light-exposed mice treated with natural combination of Rb1 and Rd or PNS at 200 mg/kg bw. Meanwhile, combined treatment of Rb1 and Rd as well as PNS did not manifest overt retinal toxicity. As shown in Supplementary Fig. S3, no significant changes in the scotopic a-wave and b-wave amplitudes were observed in mice unexposed to bright light after administration of natural combination of Rb1 and Rd, Rb1 and Rd combination at increased doses (Rb1 at 130 mg/kg bw and Rd at 45 mg/kg bw), or PNS 200 mg/kg bw. These results indicated that natural combination of Rb1 and Rd as well as PNS provided functional protection for the retinas in bright light-exposed mice.

TUNEL assay was performed to examine the impact of natural combination of Rb1 and Rd on photoreceptor apoptosis. As shown in Supplementary Fig. S4, consistent with our previous findings^24^, compared to that from vehicle-treated mice unexposed to bright light, TUNEL positivity was readily observed in a sporadic manner in the ONL in bright light-exposed vehicle-treated mice 1 d after bright light exposure. By 3 d after bright light exposure, profound increase in TUNEL positivity was observed in the ONL in bright light-exposed vehicle-treated mice. In distinct contrast, TUNEL positivity was remarkably attenuated in the ONL in bright light-exposed mice treated with natural combination of Rb1 and Rd. These results indicated that natural combination of Rb1 and Rd suppressed bright light-induced photoreceptor apoptosis in mice.

### Natural combination of Rb1 and Rd alleviated bright light-induced retinal oxidative stress

**Figure 4:**
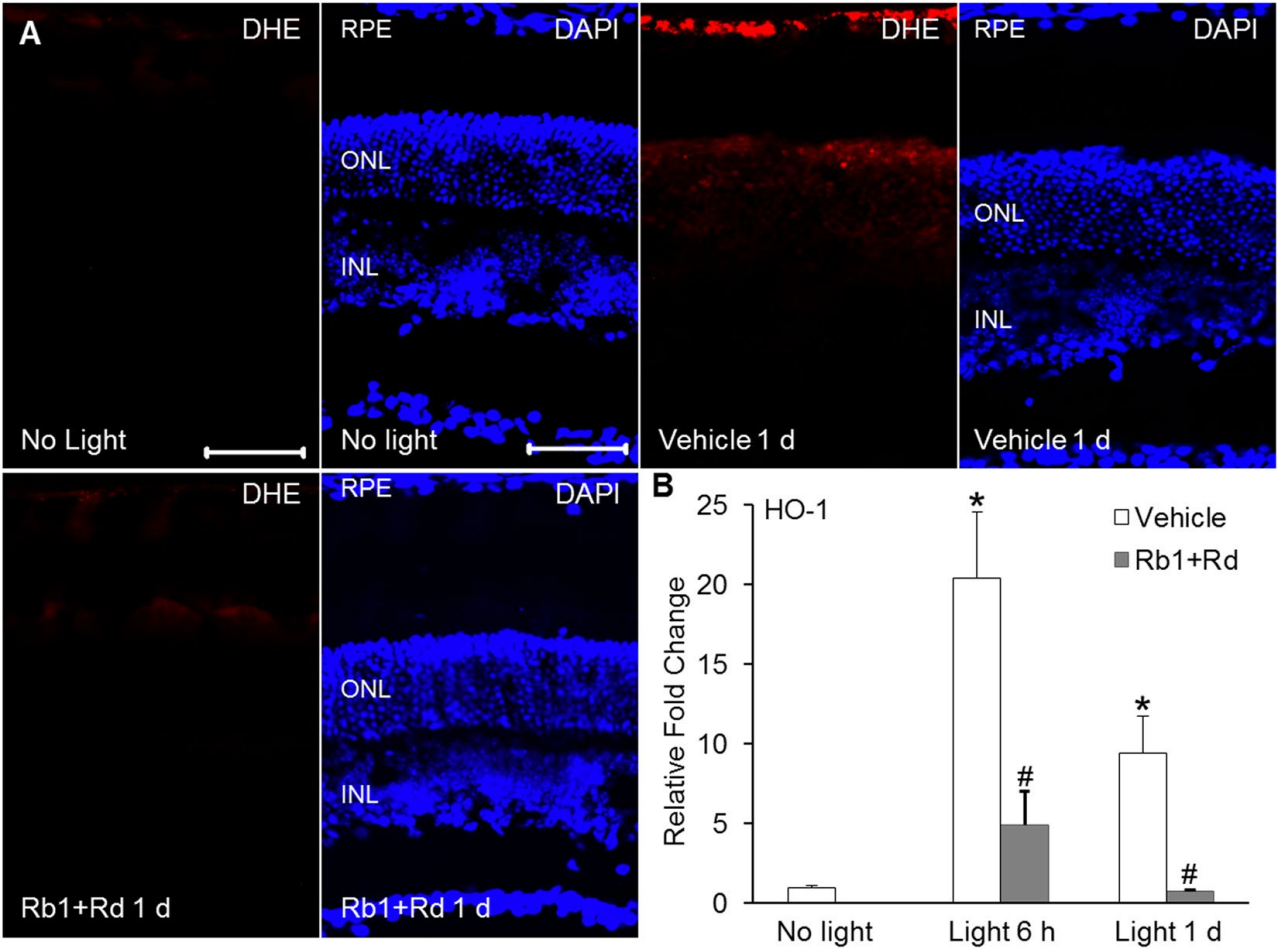
Combination of Rb1 and Rd alleviated retinal oxidative stress in bright light-exposed mice. (**A**) Retinal ROS production was examined by administering DHE to BALB/c mice unexposed to bright light (No light) and bright light-exposed mice treated with vehicle (Vehicle) or combination of Rb1 (65 mg/kg bw) and Rd (22.5 mg/kg bw) (Rb1 + Rb) (n = 4 per group). Cryosections were made from eye cups collected 1 d after illumination. ROS signals reflecting oxidized DHE (in red) and DAPI staining (in blue) were observed using a fluorescence microscope. INL, inner nuclear layer; ONL, outer nuclear layer; RPE, retinal pigment epithelium. Scale bar: 50 μm. (**B**) Retinas were isolated from vehicle-treated mice unexposed to bright light (No light) and bright light-exposed mice with the indicate treatment 6 h (Light 6 h) and 1 d (Light 1 d) after bright light exposure. Total RNA was isolated and reverse-transcribed, followed by real-time PCR analyses of the expression of HO-1 (n = 4–6 per group). Relative fold change against that from the mice unexposed to bright light (No light) was presented. The data were expressed as the mean ± S.E.M. *Compared to that from No light, *p* < 0.05; ^#^compared to that from Vehicle, *p* < 0.05.

Retinal oxidative stress was further assessed to better understand the underlying mechanism implicated in the retinal protective effects of natural combination of Rb1 and Rd. As shown in Fig. 4A, compared to that manifested by the retinas in vehicle-treated mice unexposed to bright light, production of reactive oxygen species (ROS), reflected by oxidized superoxide marker dihydroethidium (DHE), was overtly increased in the retinal pigment epithelium (RPE) of bright light-exposed vehicle-treated mice 1 d after bright light exposure. Meanwhile, ROS positivity was also readily identified in the ONL in bright light-exposed vehicle-treated mice. In contrast, ROS production in the RPE and ONL was found to be remarkably attenuated in bright light-exposed mice treated with natural combined Rb1 and Rd. Induction of heme oxygenase-1 (HO-1) has been identified to be a molecular hallmark of retinal oxidative stress in response to intense light exposure^25^. To better estimate the changes associated with oxidative stress, retinal expression of HO-1 was further analyzed. As shown in Fig. 4B, compared to that from vehicle-treated mice unexposed to bright light, significantly elevated retinal expression of HO-1 was observed in vehicle-treated mice 6 h and 1 d after bright light exposure, whereas significantly decreased expression of HO-1 was observed in bright light-exposed mice treated with natural combination of Rb1 and Rd. These results indicated that natural combination of Rb1 and Rd suppressed bright light-induced retinal oxidative stress.

### Natural combination of Rb1 and Rd attenuated bright light-induced expression of proinflammatory and cell adhesion genes in the retina

**Figure 5:**
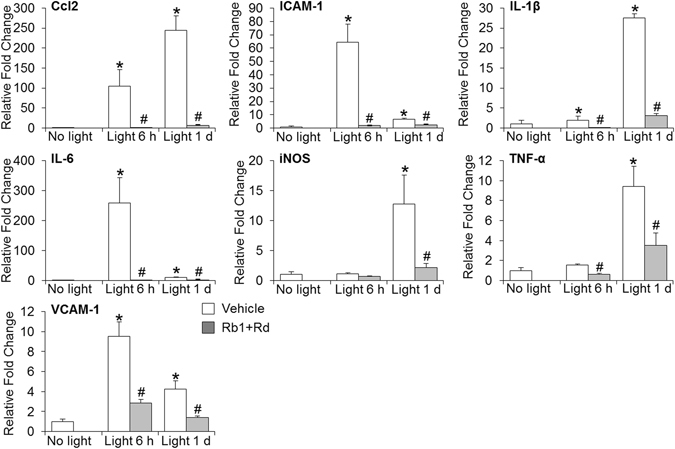
Combined treatment of Rb1 and Rd mitigated the retinal expression of proinflammatory and cell adhesion genes in bright light-exposed mice. Retinas collected from vehicle-treated mice unexposed to bright light (No light) and bright light-exposed mice treated with vehicle (Vehicle) or combination of Rb1 and Rd (Rb1 + Rd) 6 h and 1 d after illumination. Real-time PCR analyses were subsequently performed to examine the retinal expression of Ccl2, ICAM-1, IL-1β, IL-6, iNOS, TNF-α and VCAM-1 (n = 4–6 per group). Relative fold change of each gene was normalized against that from vehicle-treated mice unexposed to bright light. The data were expressed as the mean ± S.E.M. *Compared to that from No light, *p* < 0.05; ^#^compared to that from Vehicle, *p* < 0.05.

Exaggerated inflammatory response promotes the progression of retinal degeneration^7^. Induced expression of proinflammatory genes and genes encoding cell adhesion molecules associated with inflammatory responses have been noted in bright light-exposed retinas^24^. We thus examined the impact of natural combination of Rb1 and Rd on the retinal expression of genes implicated in inflammatory responses. As shown in Fig. 5, compared to that from vehicle-treated mice unexposed to bright light, remarkably elevated retinal expression of interleukin 1β (IL-1β), interleukin-6 (IL-6), chemokine (C-C motif) ligand 2 (Ccl2), intercellular adhesion molecule 1 (ICAM-1) and vascular cell adhesion molecule 1 (VCAM-1) was observed in bright light-exposed vehicle-treated mice 6 h and 1 d after bright light exposure. Moreover, significant increase in the retinal expression of inducible nitric oxide synthase (iNOS) and tumor necrosis factor-α (TNF-α) was observed in bright light-exposed vehicle-treated mice 1 d after bright light exposure. In contrast, elevated expression of these inflammatory response-associated genes triggered by bright light exposure was significantly attenuated in bright light-exposed mice treated with natural combination of Rb1 and Rd. These results suggested that natural combination of Rb1 and Rd suppressed bright light-induced inflammatory responses in the retina.

### Natural combination of Rb1 and Rd attenuated bright light-induced reactive gliosis and microglia activation in the retina

**Figure 6:**
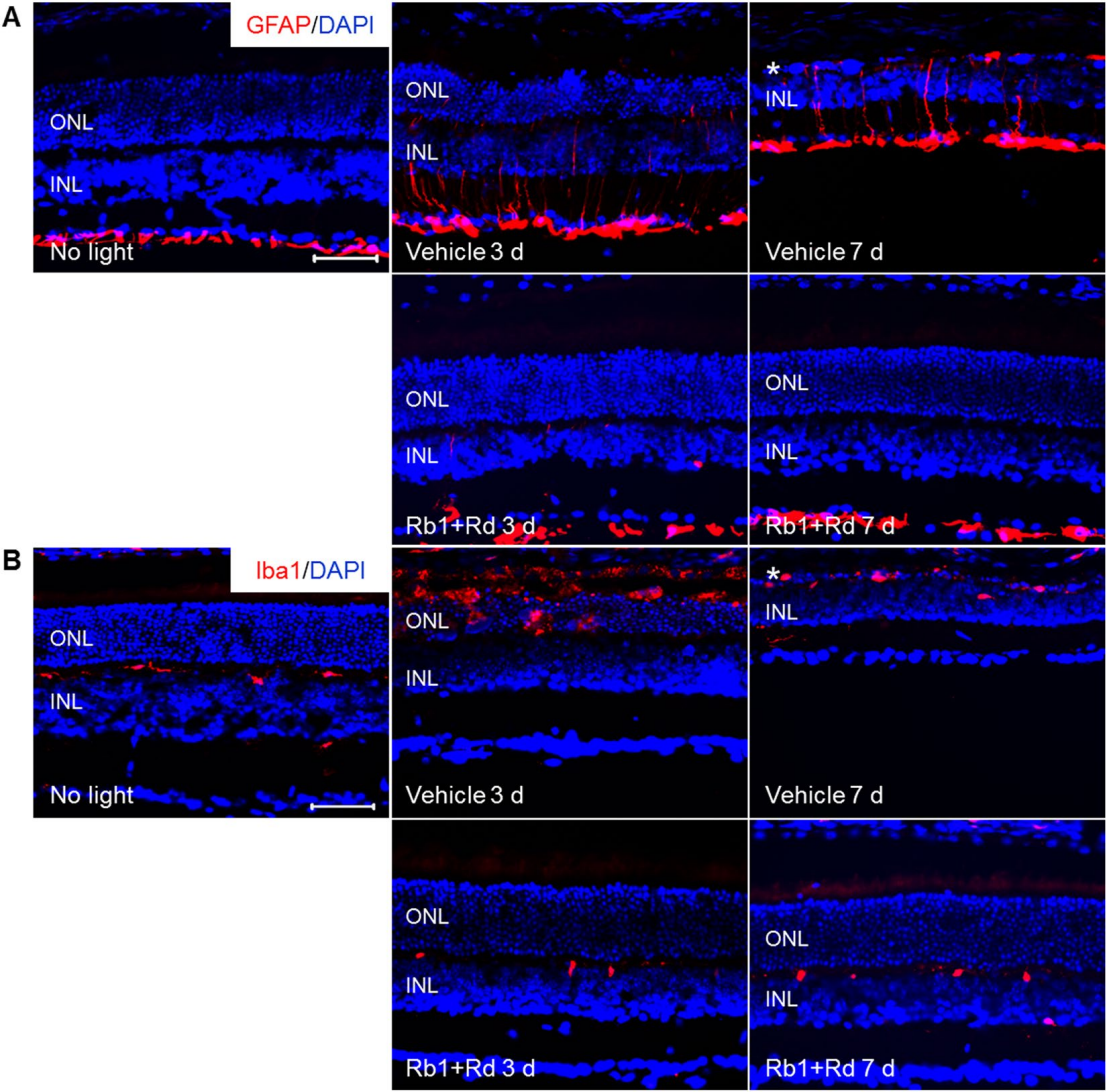
Combined Rb1 and Rd treatment attenuated reactive gliosis and microglia activation in bright light-exposed retina. Eyes were harvested from vehicle-treated mice unexposed to bright light (No light), bright light-exposed mice treated with vehicle (Vehicle) and combination of Rb1 and Rd 3 d and 7 d after illumination. Cryosections were made and subject to IHC examination of the retinal expression of GFAP (in red) (**A**) and Iba1 (in red) (**B**) (n = 4 per group). DAPI counterstaining (in blue) was performed in parallel. Asterisk indicated disrupted ONL. INL, inner nuclear layer; ONL, outer nuclear layer. Scale bar: 50 μm.

Next, the retinal expression of GFAP and Iba1, markers labeling macroglial and microglial cells, respectively, was examined to better characterize the impact of natural combination of Rb1 and Rd on reactive changes associated with retinal damage and local inflammatory response. As shown in Fig. 6A, contrary to normal expression pattern of GFAP found in nerve fiber layer (NFL) in vehicle-treated mice unexposed to bright light, expression of GFAP was readily observed not only in NFL, but also in the inner plexiform layer (IPL), inner nuclear layer (INL) and ONL 3 d and 7 d after bright light exposure, indicating hypertrophic changes of macroglial cells associated with bright light-induced retinal damage. However, this aberrant expression of GFAP was not encountered in the retinas in bright light-exposed mice treated with natural combination of Rb1 and Rd. These observations were verified by the examination of the retinal expression of vimentin, another marker for Müller cell. Enhanced expression of vimentin across the retina was readily observed in bright light-exposed vehicle-treated mice. However, similar to that observed in vehicle-treated mice unexposed to bright light, the expression of vimentin exhibited restricted pattern in bright light-exposed mice treated with natural combination of Rb1 and Rd (Supplementary Fig. S5). These results indicated that reactive gliosis caused by bright light exposure was attenuated by the treatment of naturally combined Rb1 and Rd.

Moreover, as shown in Fig. 6B, the immunoreactivity of Iba1 was observed to be restricted to the OPL in vehicle-treated mice unexposed to bright light, whereas in bright light-exposed vehicle-treated mice, Iba1 positivity was readily detected in the ONL and subretinal space 3 d after bright light exposure. Iba1 immunopositivity remained evident in severely damaged ONL in bright light-exposed vehicle-treated mice 7 d after bright light exposure. In contrast, this ectopic Iba1 expression was not observed in bright light-exposed mice treated with natural combination of Rb1 and Rd. These results demonstrated that natural combination of Rb1 and Rd suppressed bright light-induced microglia activation in the retina.

### Rb1 and Rd attenuated all-*trans*-Retinal (atRAL)-induced intracellular ROS production in ARPE 19 cells

**Figure 7:**
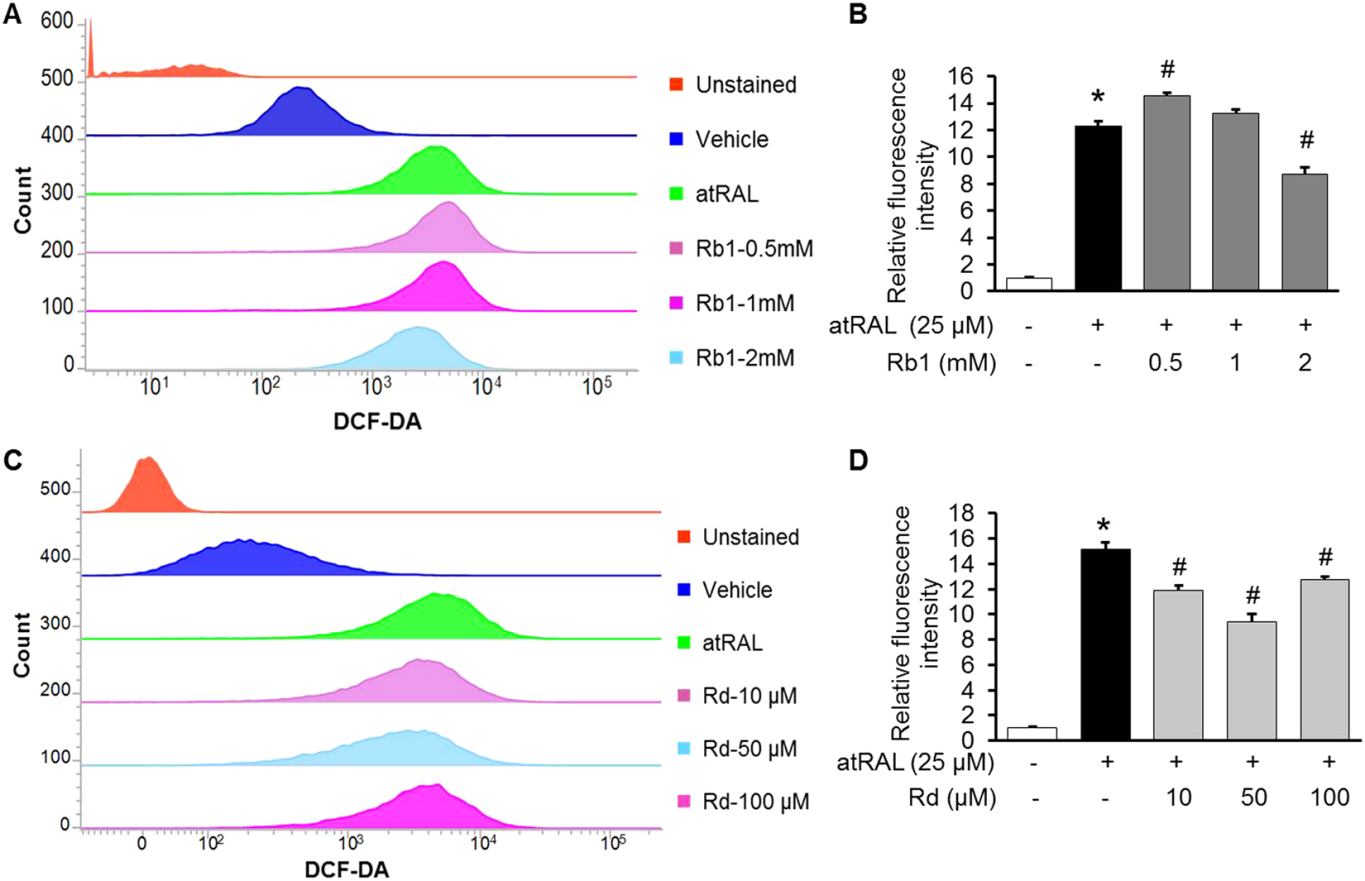
Rb1 or Rd treatment decreased atRAL-induced intracellular ROS production in ARPE19 cells. ARPE19 cells were treated with vehicle (saline or DMSO), Rb1 or Rd at the indicated concentrations for 1 h before being exposed to atRAL at 25 μM for 5 h, followed by DCF-DA staining for 30 min. After DCF-DA staining and washing, cells were resuspended and subject to further analysis by flow cytometry at the excitation and emission wavelengths of 490 nm and 520 nm, respectively. For each sample, 20,000 cells were acquired for data collection. Representative histograms of flow cytometry after DCF-DA staining were shown in (**A**,**C**). Relative DCF-DA fluorescence intensity was calculated against that from vehicle-treated cells without atRAL exposure (n = 4–5 per group) (**B**,**D**). The data were expressed as the mean ± S.E.M. *Compared to that from vehicle-treated cells unexposed to atRAL, *p* < 0.05; ^#^compared to that from atRAL-challenged vehicle-treated cells, *p* < 0.05.

As revealed in Fig. 4, natural combination of Rb1 and Rd suppressed bright light-induced oxidative stress in RPE *in vivo*. To further delineate the individual impact of Rb1 or Rd on oxidative stress, all-*trans*-Retinal (atRAL)-induced oxidative stress in human RPE-derived ARPE19 cells^5^ was further examined in the presence of Rb1 or Rd. Functional RPE cells are fully differentiated and reside in close proximity *in vivo*. Moreover, it has been noted that sub-confluent ARPE19 cells respond differently than do confluent and differentiated ARPE19 cells^26, 27^. Thus, culture condition of ARPE19 cells was first modified and the differentiated phenotype of ARPE19 cells was validated. As shown in Supplementary Fig. S6, compared to that from ARPE19 cells cultured to sub-confluency in DMEM/F12 medium supplemented with 10% FBS, increased expression of a panel of functional markers indicative of differentiated RPE cells including RPE65, UQCRC2, MERTK, TYR and SERPINF1^27^ were observed when ARPE19 cells that had reached full confluency were maintained for additional 2 d in DMEM/F12 medium supplemented with 2% FBS. The effect of Rb1 or Rd on atRAL-induced intracellular ROS generation in ARPE19 cells were therefore examined in the confluent ARPE19 cells that were cultured for 2 d in DMEM/F12 medium supplemented with 2% FBS. As shown in Fig. 7A,B, compared to that from vehicle-treated cells without atRAL incubation, significantly increased intracellular ROS production was observed in atRAL-challenged vehicle-treated cells. Rb1 treatment, however, alleviated atRAL-induced intracellular ROS production. Similar observation was made in atRAL-challenged cells treated with Rd (Fig. 7C,D). These observations supported the antioxidant activity of both Rb1 and Rd in atRAL-challenged ARPE19 cells.

### Rb1 and Rd suppressed LPS-induced expression of proinflammatory genes

**Figure 8:**
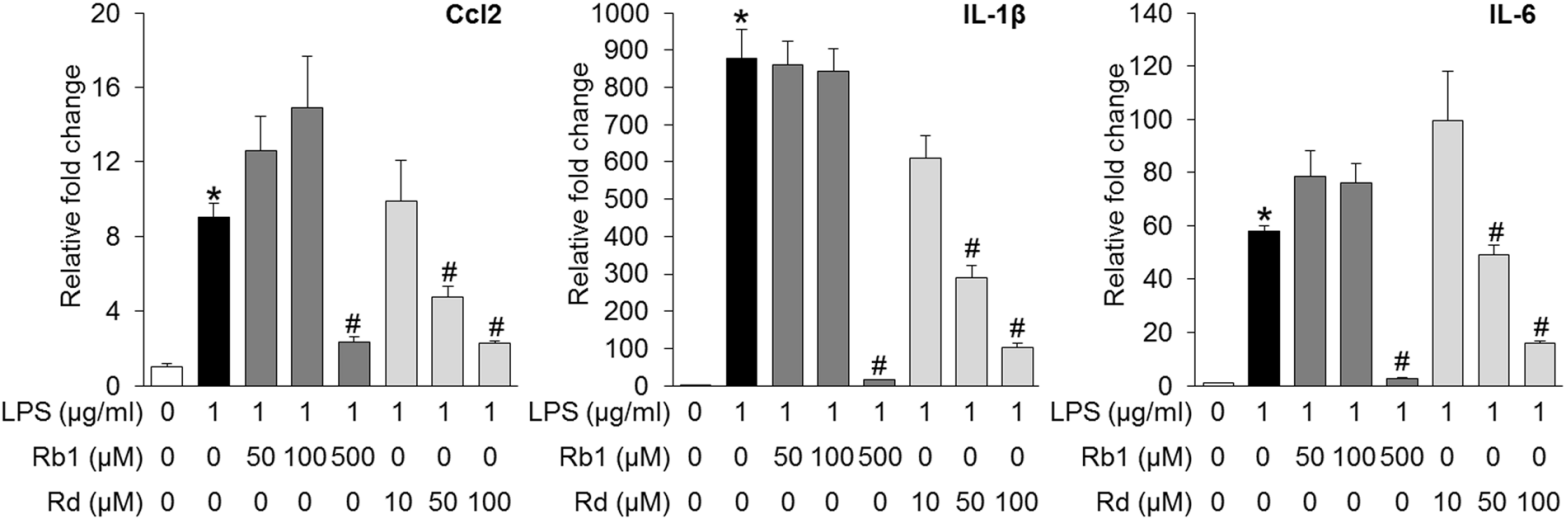
Rb1 or Rd treatment suppressed LPS-stimulated expression of proinflammatory genes in ARPE19 cells. Rb1 or Rd was applied to ARPE19 cells at the indicated concentrations 30 min before LPS stimulation delivered at 1 μg/ml for 6 h. Total RNA was extracted for analyzing the expression of Ccl2, IL-1β and IL-6 by real-time PCR (n = 4 per group). The data were expressed as the mean ± S.E.M. *Compared to that from vehicle-treated cells without LPS, *p* < 0.05; ^#^compared to that from vehicle-treated cell with LPS stimulation, *p* < 0.05.

**Figure 9:**
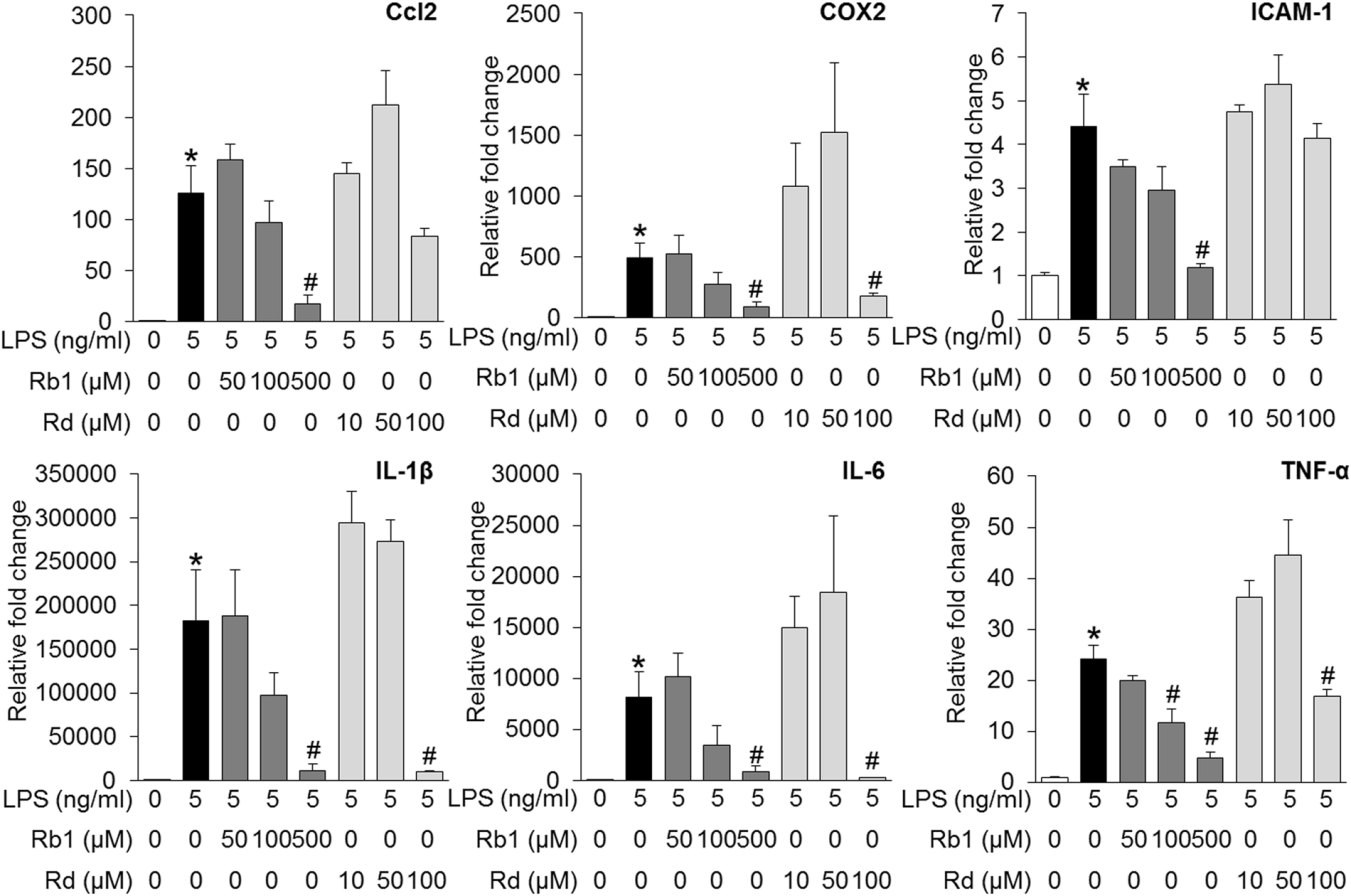
Rb1 or Rd treatment suppressed LPS-stimulated expression of proinflammatory genes in RAW264.7 cells. Rb1 and Rd were added to RAW264.7 cells at the indicated concentrations 30 min before LPS stimulation delivered at 5 ng/ml for 6 h. Cells were harvested for total RNA extraction and real-time PCR analyses to assess the expression of Ccl2, COX2, ICAM, IL-1β, IL-6 and TNF-α (n = 4 per group). The data were expressed as the mean ± S.E.M. *Compared to that from vehicle-treated cells without LPS incubation, *p* < 0.05; ^#^compared to that from vehicle-treated cell with LPS stimulation, *p* < 0.05.

Given that significantly attenuated retinal expression of proinflammatory genes was observed in bright light-exposed mice treated with natural combination of Rb1 and Rd, the individual impact of Rb1 and Rd on the expression of proinflammatory genes was further investigated. LPS-induced expression of proinflammatory genes in cultured ARPE19 cells or mouse macrophage RAW264.7 cells was assessed in the presence of Rb1 or Rd. As shown in Fig. 8, LPS-induced expression of IL-1β, IL-6 and Ccl2 in ARPE19 cells was remarkably attenuated by the treatment of both Rb1 and Rd. In RAW264.7 cells, LPS-induced expression of IL-1β, IL-6, Ccl2, COX2, ICAM-1 and TNF-α was significantly suppressed by Rb1 treatment. Similar observations were made for LPS-stimulated Rd-treated cells except for the expression of Ccl-2 and ICAM-1, which was not significantly altered by Rd treatment at the doses examined compared to that from LPS-stimulated vehicle-treated cells (Fig. 9). These results thus indicated that both Rb1 and Rd possessed anti-inflammatory activities in ARPE19 cells and RAW264.7 cells *in vitro*.

### Rb1 and Rd suppressed LPS-induced expression of proinflammatory miR-155

**Figure 10:**
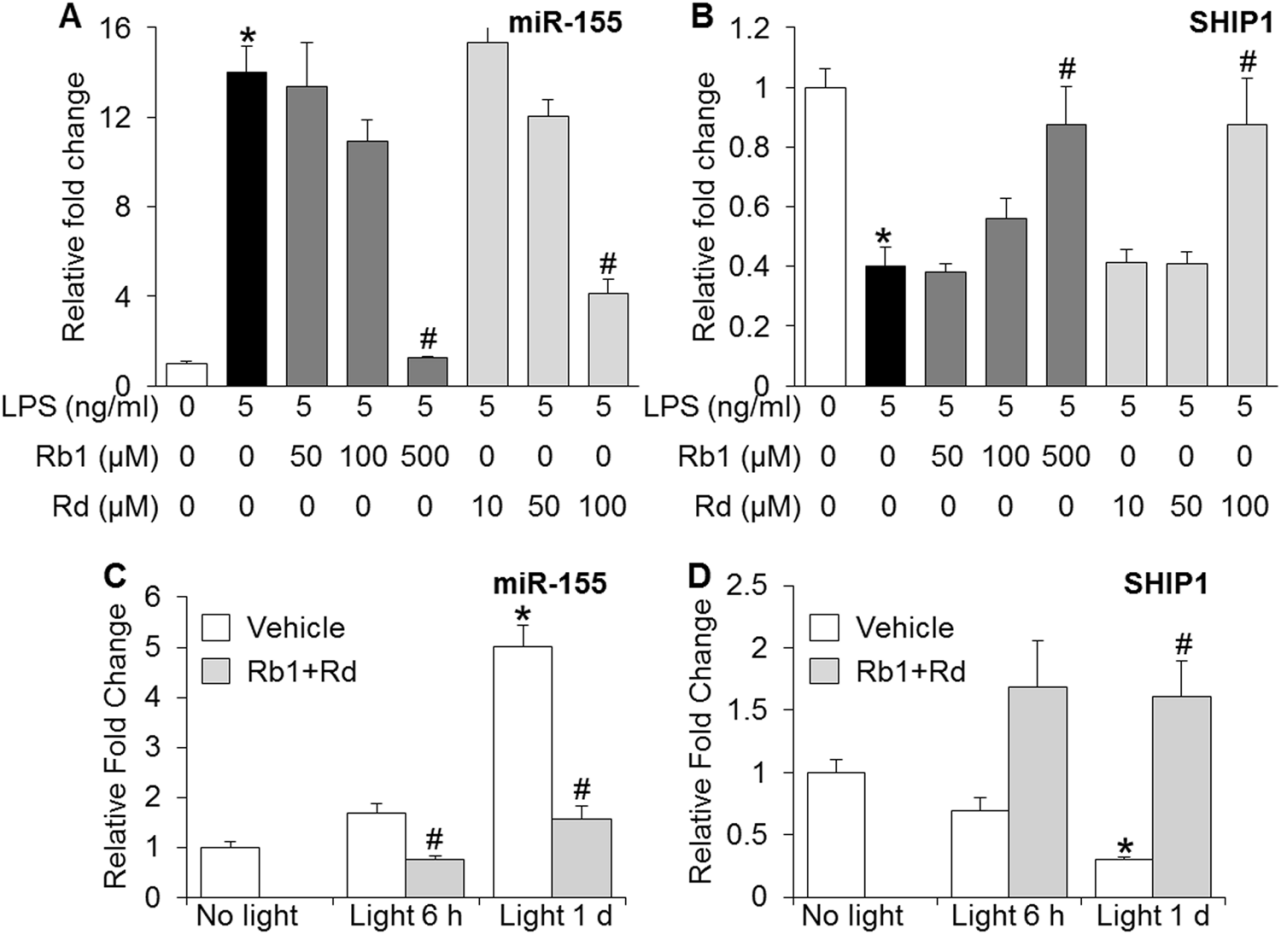
Altered expression of miR-155 and SHIP1 was implicated in the anti-inflammatory and retinal protective activities of Rb1 and Rd. A. RAW264.7 cells were first treated with Rb1 or Rd at the indicated concentrations for 30 min, followed by LPS stimulation delivered at 5 ng/ml for 6 h. Total RNA was isolated from harvested cells and real-time PCR analyses were performed to examine the expression of miR-155 (**A**) and its target gene SHIP1 (**B**) (n = 4–6 per group). Relative fold change was calculated against that detected in vehicle-treated cells without LPS stimulation. The data were expressed as the mean ± S.E.M. *Compared to that from vehicle-treated cells without LPS stimulation, *p* < 0.05; ^#^compared to that from vehicle-treated cell with LPS stimulation, *p* < 0.05. Dark-adapted BALB/c mice were treated with saline vehicle (Vehicle) or combination of Rb1 (65 mg/kg bw) and Rd (22.5 mg/kg bw) (Rb + Rd), followed by bright light exposure. Retinas were collected 6 h and 1 d after illumination along with those from vehicle-treated mice unexposed to bright light (No light). Total RNA was extracted and reverse-transcribed. Real-time PCR was subsequently performed to analyze the retinal expression of miR-155 (**C**) and SHIP1 (**D**) (n = 4–6 per group). Relative fold change of miR-155 and SHIP1 was calculated against that detected in vehicle-treated mice unexposed to bright light. The data were expressed as the mean ± S.E.M. *Compared to that from No light, *p* < 0.05; ^#^compared to that from Vehicle, *p* < 0.05.

To understand the mechanisms underlying the anti-inflammatory activities of Rb1 and Rd, the expression of miR-155, a multi-functional miRNA with primary functions in promoting proinflammatory responses^28^, was further examined. RAW264.7 cells were stimulated by LPS in the absence or presence of either Rb1 or Rd. As shown in Fig. 10A, compared to that from the cells without LPS exposure, the expression of miR-155 was increased by approximately 14 fold in LPS-stimulated vehicle-treated RAW264.7 cells. In contrast, LPS-stimulated expression of miR-155 was significantly decreased by Rb1 in a dose-dependent manner. Similar observation was made with Rd treatment in LPS-stimulated RAW264.7 cells. To further confirm the implication of miR-155 in the anti-inflammatory effects of Rb1 and Rd, the expression of Src homology 2 domain–containing inositol-5-phosphatase 1 (SHIP1), a direct target of miR-155^29^ functioning to inhibit immune cell activation^30, 31^, was also examined. As shown in Fig. 10B, a 2.5 fold decrease in the expression of SHIP1 was observed in LPS-stimulated vehicle-treated cells compared to vehicle-treated cells without LPS incubation, whereas significantly increased expression of SHIP1 was observed in LPS-stimulated cells treated with Rb1 or Rd. Collectively, these results indicated that both Rb1 and Rd were pharmacological effective at suppressing LPS-induced expression of proinflammatory miR-155 and enhancing the expression of miR-155 target, anti-inflammatory SHIP1.

### Natural combination of Rb1 and Rd treatment attenuated bright light-induced upregulation of miR-155 in the mouse retina

To further understand the biological significance of miR-155 in retinal degeneration *in vivo*, the expression of miR-155 and SHIP1 was examined in bright light-exposed retinas. As shown in Fig. 10C, compared to that from vehicle-treated mice unexposed to bright light, the retinal expression of miR-155 was increased by approximately 5 fold in bright light-exposed vehicle-treated mice 1 d after illumination. In distinct contrast, this bright light-induced retinal expression of miR-155 was significantly suppressed in bright light-exposed mice treated with natural combination of Rb1 and Rd. A significant decrease in the expression of miR-155 was also noted at 6 h after illumination in bright light-exposed mice treated with natural combination of Rb1 and Rd compared to that in bright light-exposed vehicle-treated mice. Moreover, compared to that from vehicle-treated mice unexposed to bright light, the retinal expression of SHIP1 was decreased by approximately 3 fold 1 d after bright light exposure. In contrast, compared to that from bright light-exposed vehicle-treated retinas, significantly increased retinal expression of SHIP1 was observed in bright light-exposed mice treated with natural combination of Rb1 and Rd (Fig. 10D). These results thus demonstrated that enhanced expression of miR-155 and decreased expression of SHIP1 were associated with bright light-induced retinal degeneration, whereas natural combination of Rb1 and Rd suppressed the expression of miR-155 and increased the expression of SHIP1 in bright light-exposed retinas.

## Discussion

In the current study, PNS treatment was first shown to be protective against bright light-induced retinal degeneration in mice, which was recapitulated by the treatment of natural combination of saponin components Rb1 and Rd. The natural combination of Rb1 and Rd was further shown to attenuate retinal oxidative stress, expression of proinflammatory genes, microglial activation and reactive gliosis *in vivo*. Rb1 treatment resulted in decreased level of oxidative stress in atRAL-challenged ARPE19 cells. The anti-inflammatory activities of both Rb1 and Rd were demonstrated in LPS-stimulated RAW264.7 as well as ARPE19 cells. Dysregulated expression of miR-155 and SHIP1 was revealed in bright light-exposed mouse retinas. Moreover, decreased expression of miR-155 and increased expression of SHIP1 could be implicated in the anti-inflammatory activities of Rb1 and Rd *in vitro* and retinal protective activity of natural combination of Rb1 and Rd *in vivo*.

In bright light-exposed retina, photoreceptors were preserved when natural combination of Rb1 and Rd were administered. Partial retinal protection was observed when the dose of either Rb1 or Rd was lowered from that adopted in the natural combination regimen. No significant retinal protection was observed by merely doubling the dose of either Rb1 or Rd. These results implied a synergistic effect of Rb1 and Rd on protecting photoreceptors from developing bright light-induced damage. Moreover, it is worth noting that the dosing regimens of individual and combined treatment in the current study were determined based on the natural content of each saponin compound in PNS and the effective dose of PNS. Future studies thus remain to be performed to evaluate the retinal protective activity of saponin(s) at different dosing options without considering its association of PNS.

The anti-inflammatory and antioxidant activities of Rd have been previously reported^16, 32^. Moreover, the antioxidant and anti-inflammatory effects have also been shown to be implicated in the neuroprotective effects of both Rb1 and Rd^15, 33, 34^. For instance, in addition to maintaining the balance of pro- and antioxidant mechanisms, Rd significantly attenuates the inflammatory responses in carrageenan-induced rat paw edema in part through suppressing proinflammatory ERK/JNK/NF-κB signaling^32^. Rd also significantly suppresses LPS-induced expression of iNOS and COX2 in RAW264.7 cells and ICR mouse liver through attenuating NF-κB activation^16^. The therapeutic effects of Rd against cerebrovascular ischemia-reperfusion injuries as well as neuronal loss in Parkinson’s disease model have also been reported^18, 35^. Meanwhile, Rb1 is pharmacologically active in immunoregulation^14^ and suppression of inflammatory responses^13, 34^. Mitigated NF-κB signaling is noted to be involved in the anti-inflammatory effects of Rb1 in protecting against cerebral ischemic injuries^34^. However, the pharmacological implication of the anti-inflammatory and antioxidant activities of Rb1 and Rd in retinal protection has been revealed prior to our current study.

Oxidative stress plays a primary role in the pathogenesis of retinal degeneration^36–38^. As an early event in the development of AMD, oxidative stress contributes to RPE dysfunction that promotes the loss of photoreceptors during disease progression^39, 40^. In the current study, significantly attenuated oxidative stress was observed in bright light-exposed mice treated with naturally combined Rb1 and Rd. In cultured ARPE19 cells *in vitro*, both Rb1 and Rd could partially attenuate atRAL-induced overproduction of intracellular ROS. Given significant implication of oxidative stress in retinal degeneration, attenuation of retinal oxidative stress could in part account for the retinal protective effects of natural combination of Rb1 and Rd.

Inflammation has been increasingly acknowledged as an essential component of the vicious cycle that promotes progressive degeneration of neuronal tissue under disease settings^41^. In the damaged retina, both Müller glial cells and microglial cells contribute to inflammatory responses. Reactive Müller gliosis occurs in virtually every retinal disease. In the adult mammalian retina, Müller glial cells undergoing gliotic changes become a source of proinflammatory factors and promote further degeneration^42, 43^. Moreover, in response to stimuli such as neuronal death, enhanced activation of microglial occurs^44^. Activated microglial cells produce increased amount of proinflammatory cytokines and chemokines such as IL-1, TNF-α and CCL2 that aggravate neuronal death and propagate inflammatory responses. For instance, CCL2 regulates microglial migration leading to presence and accumulation of activated microglial at the site of neuronal injury^45^. Suppressing microglial activation protects against photoreceptor loss caused by light damage^46–48^. Microglial activation has therefore been established as hallmark pathology as well as therapeutic target in degenerative retinas. As shown in the present study, natural combination of Rb1 and Rd led to significantly decreased expression of proinflammatory cytokines and chemokines such as IL-1β, IL-6, TNF-α and CCL2 in bright light-exposed retinas. Meanwhile, attenuated retinal reactive gliosis as well as microglial activation were observed as a result of combined treatment of Rb1 and Rd. Moreover, although consistent with previous reports, anti-inflammatory activity was exhibited by Rb1 and Rd in RAW264.7 cells, it was not limited to RAW264.7 cells, anti-inflammatory activity of Rb1 and Rd was also observed in ARPE19 cells challenged by LPS. Given that exaggerated inflammatory responses aggravate tissue damage, which is of particular relevance to the deterioration of neuronal tissue such as retina that lacks regenerative capacity^49^, the anti-inflammatory activities of Rb1 and Rd may contribute to their therapeutic potential of retinal protection.

Moreover, elevated expression of miR-155 in bright light-exposed retinas and the implication of miR-155 in the anti-inflammatory activities of Rb1 and Rd were noted in the current study. miR-155 is known as a common proinflammatory mediator shared by different inflammatory mechanisms, functioning as an important component of the proinflammatory cascade in immune cells. Elevated level of miR-155 contributes to neuroinflammation and inhibition of miR-155 is neuroprotective^50^. miR-155 has thus been valued as a promising target for modulating neuroinflammatory responses^50, 51^. One of the direct targets of miR-155 in immune cells is SHIP1^29^. Through modulating PI3K signaling pathway, SHIP1 functions to downregulate the activation and migration of immune cells and thus mitigate inflammatory responses^52^. It is noted that pharmacological agents activating SHIP1 can attenuate the function and migration of immune cells and thus exhibit anti-inflammatory effects^53–55^. Although the pro-inflammatory function of miR-155 has been well recognized, the implication of miR-155 in retinal degenerative disorders remains to be addressed. As shown in the current study, bright light exposure induced microglial activation and increased the expression of a panel of proinflammatory cytokines. Meanwhile, significantly elevated expression of miR-155 and decreased expression of its target SHIP1 were observed in bright light-exposed retinas, suggesting for the first time that elevated expression of miR-155 could be involved in the inflammatory responses associated with bright light-induced retinal degeneration. Further studies are required to clarify the underlying mechanisms for bright light-induced retinal expression of miR-155. Moreover, the implication of miR-155 in the inflammatory changes during retinal degeneration remains to be explored in depth in future studies. It is worth noting that both Rb1 and Rd were pharmacologically active in suppressing the expression of miR-155 and increasing the expression of SHIP1 in LPS-challenged RAW264.7 cells. These observations added new layer of understanding of the anti-inflammatory activities of Rb1 and Rd in immune cells. However, detailed mechanisms underlying the modulatory effects of Rb1 and Rd on the expression of miR-155 and SHIP1 require clarification in the future studies. Furthermore, natural combination of Rb1 and Rd resulted in significantly decreased expression of miR-155 and increased expression of SHIP1 in bright light-exposed retinas. Given that natural combination of Rb1 and Rd suppressed the retinal expression of proinflammatory genes and activation of macroglial and microglial cells in bright light-exposed retinas and the important function of miR-155 in regulating neuroinflammation, it is possible that attenuated expression of miR-155 and enhanced expression of SHIP1 are involved in the retinal protection conferred by the natural combination of Rb1 and Rd in bright light-exposed mice. Taken together, our data provided novel insights into the mechanisms and biological significance of the anti-inflammatory properties of Rb1 and Rd.

In conclusion, the present work not only demonstrated the retinal protective effect of PNS, but also identified that both Rb1 and Rd were required for the photoreceptor protective activity of PNS. The experimental evidence presented here justified combination of Rb1 and Rd to be further evaluated as a novel treatment for retinal degenerative disorders.

## Methods

### Chemicals

PNS was generously provided by BioAsia International Life Science Research Limited (Shanghai, China). Major saponin compounds present in PNS and their respective content were previously described^23^, including ginsenoside Rb1 (Rb1) (32.32%), ginsenoside Rg1 (Rg1) (25.38%), ginsenoside Rd (Rd) (11.17%) and notoginsenoside R1 (R1) (8.88%). Individual saponin compound such as Rb1, Rg1, Rd and R1 was purchased from Shanghai Yuanye Biological Technology Co. LTD (Shanghai, China). The purity of each compound was >98%. Sterile saline (0.9%) was used to dissolve PNS, Rb1 and Rg1. Rd, R1 and all the indicated combinatorial treatments were prepared using 30% DMSO.

### Cell culture

Mouse RAW264.7 cells were purchased from Shanghai Institute of Biological Science (SIBS, China) and cultured in Dulbecco’s Modified Eagle Medium (DMEM) (Gibco, Thermo Fisher Scientific, USA) supplemented with 10% fetal bovine serum (FBS), 50 μg/ml streptomycin, and 50 U/ml penicillin (Gibco, Thermo Fisher Scientific, USA). Human ARPE19 cells were obtained from American Type Culture Collection (ATCC, USA) and were cultured in DMEM and Nutrient Mixture F-12 (DMEM/F-12) medium (Gibco, Thermo Fisher Scientific, USA) supplemented with 10% or 2% FBS, 50 μg/ml streptomycin, and 50 U/ml penicillin (Gibco, Thermo Fisher Scientific, USA).

### Lipopolysaccharides (LPS) stimulation

Rb1 was applied to ARPE19 or RAW264.7 cells at 50, 100, and 500 μM. ARPE19 or RAW264.7 cells were incubated with Rd at 10, 50, 100 μM. After 30 min of pre-incubation with indicated compound, ARPE 19 and RAW264.7 cells were stimulated with LPS (Sigma-Aldrich, USA) at the concentration of 1 μg/mL and 5 ng/mL, respectively. Cells were collected for gene expression analyses 6 h after LPS stimulation.

### *In vitro* detection of intracellular ROS generation by flow cytometry

ARPE19 cells maintained in DMEM/F-12 medium supplemented with 10% FBS were seeded in 24-well plates at the number of 1.5 × 10^5^ cells per well. When cells reached full confluence, the culture medium was replaced by DMEM/F-12 supplemented with 2% FBS. Cells were then further cultured for 2 d before the treatment of Rb1 or Rd at the indicated concentrations. After incubation of Rb1 or Rd for 1 h, atRAL (Sigma-Aldrich, USA) was applied at 25 μM for 5 h. ROS probe 2′, 7′-dichlorofluorescein diacetate (DCF-DA) (Sigma-Aldrich, USA) was then added to ARPE19 cells at 1 µM and incubated at 37 °C in the dark for 30 min. After DCF-DA staining, cells were resuspended in 1 mL phosphate-buffered saline and analyzed by flow cytometry (FACSVerse, BD Biosciences, USA) at the excitation and emission wavelengths of 490 nm and 520 nm, respectively. Same number of cells (20,000) was applied to each sample for data acquisition. DCF-DA fluorescence intensity was analyzed using FACSuite (BD Biosciences, USA).

### Mouse treatment

Four to five-week-old Female BALB/c mice were obtained from the Shanghai Laboratory Animal Research Center. Mice were maintained at 23 ± 2 °C under a 12 h light/dark cycle and allowed free access to food and water. For mice subject to bright light exposure, dark adaptation was carried out for 24 h prior to illumination delivered at 10,000 lux for 30 min (compact fluorescence lamp, 45 W, Chaoya Lighting, Shanghai, China). All of drug treatments were administered 30 min before bright light exposure via intraperitoneal injection at indicated doses. PNS, dissolved in saline, was administered at the dose of 50 and 200 mg/kg body weight (bw). The dose of Rb1, Rg1, Rd and R1 for mono and combinatorial treatments was calculated based on their respective content in PNS with reference to PNS treatment given at 200 mg/kg bw, which were indicated as following: Rb1, 65 mg/kg bw; Rg1, 50 mg/kg bw; Rd, 22.5 mg/kg bw and R1, 18 mg/kg bw. Additional experiments were performed with Rb1 administered at the dose of 130 mg/kg bw and Rd at 45 mg/kg bw. Combination of Rb1 and Rd was also examined by additional dosing regimens, which included Rd at a constant dose of 22.5 mg/kg bw and Rb1 at the dose of 44 mg/kg bw (Rd + Rb1L1) or 22 mg/kg bw (Rd + Rb1L2) as well as Rb1 at a constant dose of 65 mg/kg bw and Rd at the dose of 15 mg/kg bw (Rb + RdL1) or 7.5 mg/kg bw (Rb1 + RdL2). All mouse care and experimental procedures were approved by the Institutional Animal Care and Use Committee of Shanghai University of TCM and carried out in adherence to the ARVO Statement for the Use of Animals in Ophthalmic and Vision Research.

### Optical coherence tomography (OCT)

OCT (Optoprobe, Canada) was used for *in vivo* imaging of mouse retinas 7 days after bright light exposure. Briefly, mice were anesthetized by intraperitoneal injection of pelltobarbitalum natricum at the dose of 65 mg/kg bw and pupils were dilated by 1% tropicamide before OCT imaging.

### Electroretinogram (ERG)

Dark-adapted mice were anesthetized with a mixture of ketamine hydrochloride (82.5 mg/kg bw) and xylazine (8.25 mg/kg bw). Scotopic ERGs were generated with flashes of green light at the intensities ranging from −2 log cd·s·m^−2^ to 3.1 log cd·s·m^−2^. Five recordings were made at sufficient intervals between flash stimuli to allow recovery from any photobleaching effects. ERG was recorded under dim red light and analyzed with the universal testing and electrophysiological system, Ganzfeld (Phoenix Research labs, USA).

### Histology and immunohistochemistry (IHC)

Enucleated eyes or eye cups free of cornea and lens were fixed in 4% paraformaldehyde before further processing to make paraffin sections or cryosections. Paraffin sections 4 μm thick were subject to hematoxylin and eosin (H&E) staining. The H&E-stained sections were observed and the images were recorded by a fluorescent microscope under bright field settings (DM6000B, Leica, Germany). IHC of rhodopsin (1:1000, Novusbio, USA) and opsin M (1:100, Millipore, USA) was performed on paraffin sections 4 μm thick as well. IHC of glial fibrillary acidic protein (GFAP) (1:500, Dako, Denmark), vimentin (1:50, Cell Signaling Technology, USA) and Iba1 (1:500, Wako, Japan) was performed on cryosections 12 um thick. Counterstaining of 4-6-Diamidino-2-phenylindole (DAPI) was performed for the indicated IHC assessment. The thickness of outer nuclear layer (ONL) was measured after H&E or DAPI staining. Images were recorded using a fluorescent microscope under fluorescent settings (DM6000B, Leica, Germany).

### TdT-mediated dUTP nick-end labeling (TUNEL) assay

Eye cups were made from enucleated eyes collected from mice unexposed to bright light and bright light-exposed mice with the indicated treatment 24 h and 72 h after illumination. Cryosections 12 μm thick were then made and subject to TUNEL assay following the manufacturer’s instructions (DeadEnd™ Fluorometric TUNEL system, Promega, USA). Images were observed using a fluorescent microscope (DM6000B, Leica, Germany).

### Real-time PCR analysis

Mouse retinas were collected from mice unexposed to bright light and light-exposed mice with indicated treatment 6 h and 24 h after illumination, respectively. Total RNA was extracted using the TRIzol reagent (Invitrogen, USA). ARPE19 or RAW264.7 cells were harvested for total RNA extraction 6 h after LPS stimulation. The PrimeScript RT Master Mix (TaKaRa, Japan) and miScript II RT kit (Qiagen, Germany) were used for reverse transcription of mRNAs and miRNAs, respectively. Triplicate real-time PCR reactions were performed for each template using the QuantiTect SYBR Green PCR Master Mix (Qiagen, Germany) and run on Roche Light Cycler 480 II. Fold changes of the expression of indicated genes or miR-155 were calculated according to 2^−[Ct(candidate)−Ct(internal control)]^. GAPDH and RUN6B were included as internal controls for the expression of genes and miR-155, respectively. The primer sequences (5′-3′) for gene expression analyses were: mouse Ccl2: forward, AGCTGTAGTTTTTGTCACCAAGC, reverse: GTGCTGAAGACCTTAGGGCA; mouse COX2: forward, CCGTACACATCATTTGAAGAACTTA, reverse, CTACCATGGTCTCCCCAAAGAT; mouse ICAM-1: forward, TCCGGACTTTCGATCTTCCAGCTAC, reverse, CCAGGTATATCCGAGCTTCAGAGGC; mouse IL-1β: forward, TGCCACCTTTTGACAGTGATG, reverse, AAGGTCCACGGGAAAGACAC; mouse IL-6: forward, CCAAGAACGATAGTCAATTCCAGAA, reverse, AAGAAGGCAACTGGATGGAAGT; mouse iNOS: forward, ATAGTTTCCAGAAGCAGAATGTGAC, reverse, AGGACATAGTTCAACATCTCCTGGT; mouse SHIP1: forward, GAGGACGATAAATTCACTGTTCAG, reverse, TTTCCTTCTTGTAAAAGTCGATGAG; mouse TNF-α: forward, ACGTCGTAGCAAACCACCAA, reverse, GCAGCCTTGTCCCTTGAAGA; mouse VCAM-1: forward, AAGAAAGGGAGACTGTCAAAGAACT, reverse, AACTTCATTATCTAACTTCCTGCCC; mouse GAPDH: forward, CCGGTGCTGAGTATGTCGT, reverse, CCTTTTGGCTCCACCCTTC; human Ccl2: forward, CTCATAGCAGCCACCTTCATTC, reverse, CTCTGCACTGAGATCTTCCTATTG; human ICAM-1: forward, GAATCAGTGACTGTCACTCGAGAT, reverse, CCACAGTGATGATGACAATCTCATA; human IL-1β: forward, TTATTACAGTGGCAATGAGGATGAC, reverse, GGAAGGAGCACTTCATCTGTTTAG; human TNF-α: forward, CCTCTCTCTAATCAGCCCTCTG, reverse, CTACAACATGGGCTACAGGCTT; human RPE65: forward, AAAGAAGGACATGTCACATACCACA, reverse, GGAAAATATATTCTTGCAGGGATCT; human UQCRC2: forward, CTCAGGACCTTGAGTTTACCAAGT, reverse, GCTGAAGTCCTCATATCTACTGCC; human MERTK: forward, AAGTTACAGCAATAATCGCTTCCT, reverse, TGTACTTCGATGTAGATGGGATCA; human KRT18: forward, CTTGCTGCTGATGACTTTAGAGTC, reverse, CTTGAGAGCCTCGATCTCTGTC; human TYR: forward, ATCATTCTTCTCCTCTTGGCAGA, reverse: GTTCTGGATTTGTCATGGTTTCCA; human SERPINF1: forward, GATGAGATCAGCATTCTCCTTCTC, reverse, ATAGCGTAAAACAGCCTTAGGGT; human β-actin: forward, CCAGCTCACCATGGATGATGAT, reverse, ACATAGGAATCCTTCTGACCCAT; human GAPDH: forward, ACTCTGGTAAAGTGGATATTGTTGC, reverse, GGAATCATATTGGAACATGTAAACC. The primers sequences (5′-3′) for miRNA expression analyses were miRNA universal primer: GATTGAATCGAGCACCAGTTAC; mmu-miR-155, TTAATGCTAATTGTGATAGGGGT and RUN6B, ACGCAAATTCGTGAAGCGTT.

### *In vivo* detection of ROS

Dihydroethidium (DHE) (Sigma-Aldrich, USA) was intraperitoneally administered to mice at the dose of 20 mg/kg bw for the detection of ROS production *in situ*. Mice were euthanized and eyes were enucleated 2 h after DHE administration, followed by fixation in 4% paraformaldehyde and processing for cryosectioning. Cryosections 12 μm thick were subject to DAPI staining, followed by assessment for red fluorescence indicative of oxidized dihydroethidium using a fluorescent microscope (DM6000B, Leica, Germany).

### Statistical analysis

Data were expressed as the mean ± standard error of mean (S.E.M.). Statistical analyses were performed using one-way ANOVA and independent samples t-test (SPSS 18, USA). Statistical significance was defined by *p* < 0.05.

## Electronic supplementary material

Supplementary Information
