# Supplementary material for: Combination of ginsenoside Rb1 and Rd protects the retina against bright light-induced degeneration
Source: Sci Rep. 2017 Jul 20;7:6015. doi: 10.1038/s41598-017-06471-x (PMC5519667; doi:10.1038/s41598-017-06471-x)

Supplementary Information

Combination of ginsenoside Rb1 and Rd protects the retina against bright light-induced degeneration

Minjuan Bian [1], Xiaoye Du [1], Peiwei Wang [1], Jingang Cui [1], Jing Xu [2], Jiangping Gu [2], Teng Zhang [1]*, Yu Chen [1]*

Affiliation: [1] Yueyang Hospital and Clinical Research Institute of Integrative Medicine, Shanghai University of Traditional Chinese Medicine, Shanghai 200437, China; [2] Department of Pharmacy, East China University of Science and Technology, Shanghai 201203, China.

* To whom correspondence should be addressed: Shanghai University of Traditional Chinese Medicine, 110 Ganhe Rd, Shanghai 200437, China. Tel.: (86)21-65161782 Ext 2048; Fax: (86)21-65161782 Ext 6519; E-mail: chenyu6639@hotmail.com; and Tel.: (86)21-65161782 Ext 6159; Fax: (86)21-65161782 Ext 6519; E-mail: zhangteng501@hotmail.com.

Supplementary Information

Supplementary Figure Legends

Supplementary Fig. S1. The effect of PNS and major saponin components of PNS on bright light-induced retinal degeneration. Thirty minutes before bright light exposure, dark-adapted BALB/c mice were treated with 30% DMSO vehicle (Vehicle), PNS at 50 mg/kg bw (PNSL), PNS at 200 mg/kg bw (PNSH), Rb1, Rg1, Rd and R1 at the dose of 65, 50, 22.5 and 25 mg/kg bw, respectively, as well as the indicated combinations with each saponin component administered at the same dose as that used in the mono treatment. Rb1 and Rd were also examined at an increased dose of 130 mg/kg bw (Rb1H) and 45 mg/kg bw (RdH), respectively. Bright light exposure was then applied at the intensity of 10,000 lux for 30 min. OCT imaging was performed 7 d after illumination (n=4 per group). Asterisk indicated disrupted ONL. INL, inner nuclear layer; ONL, outer nuclear layer.

Supplementary Fig. S2. Combination of Rb1 and Rd administered at different dosing regimens resulted in partial protection against bright light-induced retinal degeneration in mice. Four combinatorial dosing regimens were assessed in bright light-exposed BALB/c mice, which included Rb+RdL1 (Rb1, 65 mg/kg bw and Rd, 15 mg/kg bw), Rb+RdL2 (Rb1, 65 mg/kg bw and Rd, 7.5 mg/kg bw), Rd+Rb1L1 (Rd, 22.5 mg/kg bw and Rb1, 44 mg/kg bw) and Rd+Rb1L2 (Rd, 22.5 mg/kg bw and Rb, 22 mg/kg bw). **A**. OCT imaging was carried out 7 d after bright light exposure at 10,000 lux for 30 min (n=4-6 per group). **B**. Histological examination of retinal morphology was performed after OCT imaging (n=3-6 per group). **C**. ONL thickness was measured at 500 μm from optic nerve head in the superior retina. Asterisk indicated disrupted ONL. INL, inner nuclear layer; ONH, optic nerve head; ONL, outer nuclear layer. Scale bar: 50 μm. The

data were expressed as the mean±S.E.M (n=3-6 per group). * Compared to that from No light, $p<0.05$; # Compared to that from Vehicle, $p<0.05$.

Supplementary Fig. S3. No overt retinal toxicity was associated with the treatment of PNS or Rb1 and Rd combination. BALB/c mice were treated with 30% DMSO vehicle (Vehicle), PNS at 200 mg/kg bw, natural combination of Rb1 and Rd (Rb1+Rd-L) or increased doses of Rb1 (130 mg/kg bw) and Rd (45 mg/kg bw) (Rb1+Rd-H). Retinal function was examined 24 h later. Scotopic ERGs were recorded and amplitudes of a-wave (**A**) and b-wave (**B**) were plotted. Data were expressed as mean±S.E.M (n=4-5 per group).

Supplementary Fig. S4. Combined treatment with Rb1 and Rd suppressed bright light-induced photoreceptor apoptosis. Dark-adapted BALB/c mice were treated with 30% DMSO vehicle (Vehicle) or combination of Rb1 (65 mg/kg bw) and Rd (22.5 mg/kg bw) for 30 min and then exposed to bright light at 10,000 lux for 30 min. BALB/c mice unexposed to bright light were treated with saline vehicle (No light). Cryosections were made from eye cups collected 1 d and 3 d after illumination along with those from the mice unexposed to bright light. TUNEL staining (in green) and DAPI counterstaining (in blue) were performed to assess apoptosis (n=4 per group). INL, inner nuclear layer; ONL, outer nuclear layer; RPE, retinal pigment epithelium. Scale bar: 50 μm.

Supplementary Fig S5. Combination of Rb1 and Rd suppressed bright light-induced vimentin expression in the retina. Dark-adapted BALB/c mice were treated with 30% DMSO vehicle (Vehicle) or combination of Rb1 (65 mg/kg bw) and Rd (22.5 mg/kg bw) for 30 min before bright light exposure at 10,000 lux for 30 min. Eye cups were then made from the vehicle-treated mice unexposed to bright light (No light) and the mice

with the indicated treatment 3 d and 7 d after bright light exposure. Cryosections of eye were subject to IHC of vimentin expression (in red) and DAPI counterstaining (in blue) (n=4 per group). Asterisk indicated disrupted ONL. INL, inner nuclear layer; ONL, outer nuclear layer. Scale bar: 50 μm.

Supplementary Fig S6. The morphology of ARPE19 cells and the expression of signature genes identifying functional RPE in ARPE19 cells under different culture conditions. ARPE19 cells were seeded into 24-well plates at the number of 1.5×10^5 cells per well and cultured in DMEM/F-12 medium supplemented with 10% FBS until reaching full confluence. DMEM/F-12 medium supplemented with 2% FBS was then adopted to culture ARPE19 cells for additional 3 d. **A.** Bright field micrographs were taken to document the morphology of ARPE19 cells that were sub-confluent (Sub), full confluent (Full), and confluent ARPE19 cells cultured in DMEM/F-12 medium supplemented with 2% FBS for 2 d (2 d). Scale bar: 300 μm. **B.** Total RNA was isolated from sub-confluent ARPE19 cells (Sub), confluent ARPE19 cells (Full), confluent ARPE19 cells cultured in DMEM/F12 medium supplemented with 2% FBS for 1 d (1 d), 2 d (2 d) and 3 d (3 d). After reverse transcription, real-time PCR analyses were performed to examine the expression of signature genes identifying mature functional RPE. The expression of β-actin was analyzed in parallel to serve as an internal control. Relative fold change against that from sub-confluent cells was presented. Data were expressed as mean±S.E.M (n=4 per group). ***** Compared to that from sub-confluent cells (Sub), $p<0.05$.

Supplementary Information

Supplementary Figures

Supplementary Fig. S1

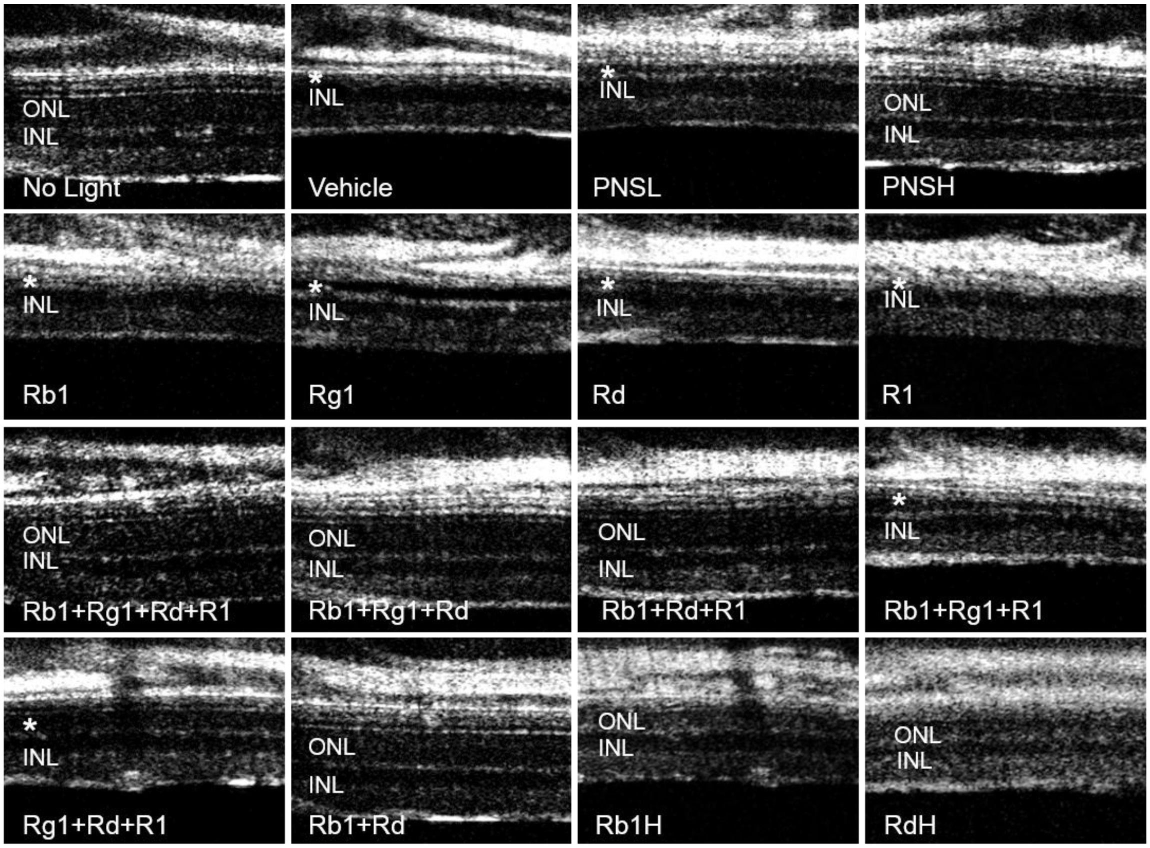

Supplementary Fig. S2

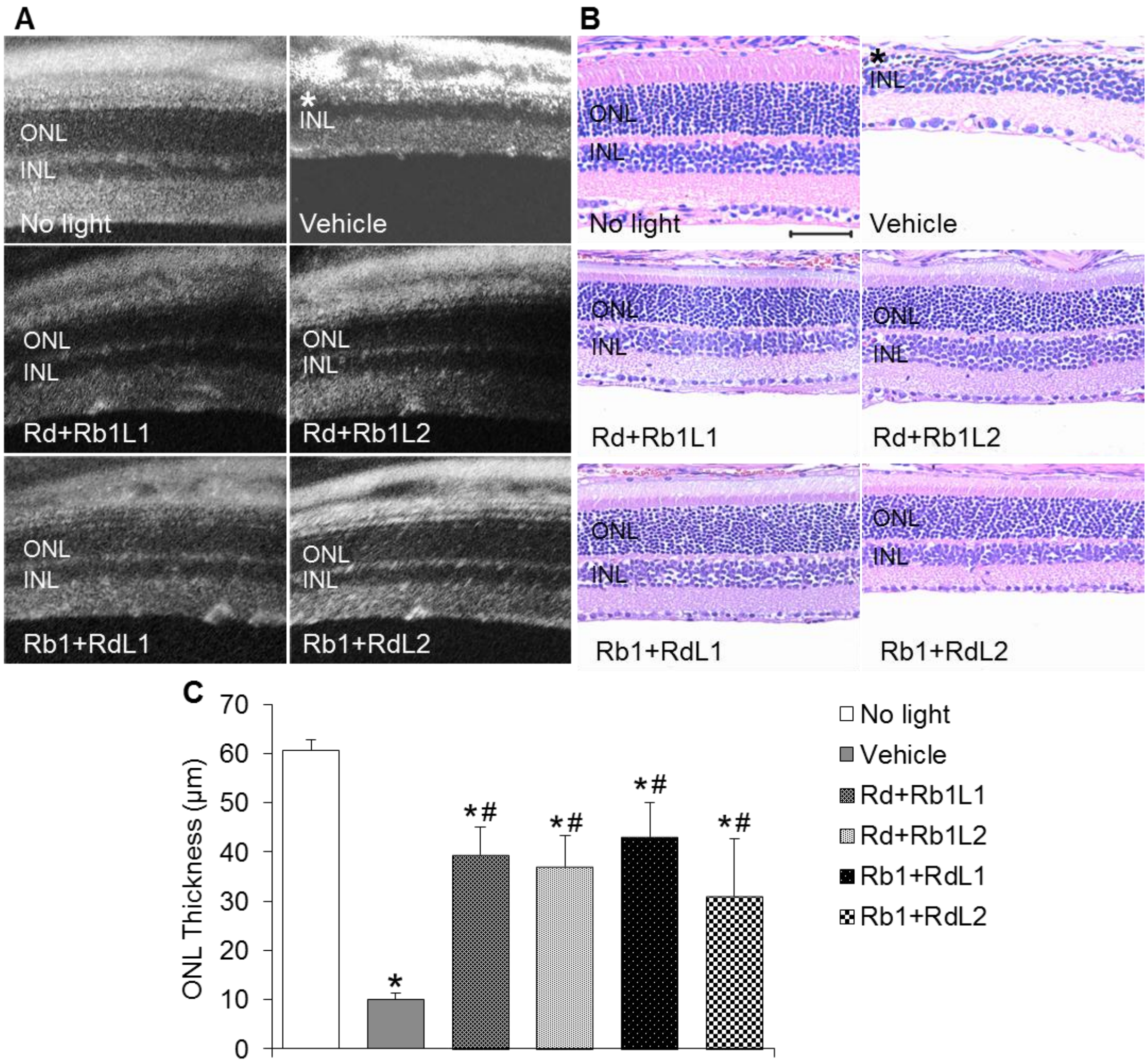

Supplemental Fig. S3

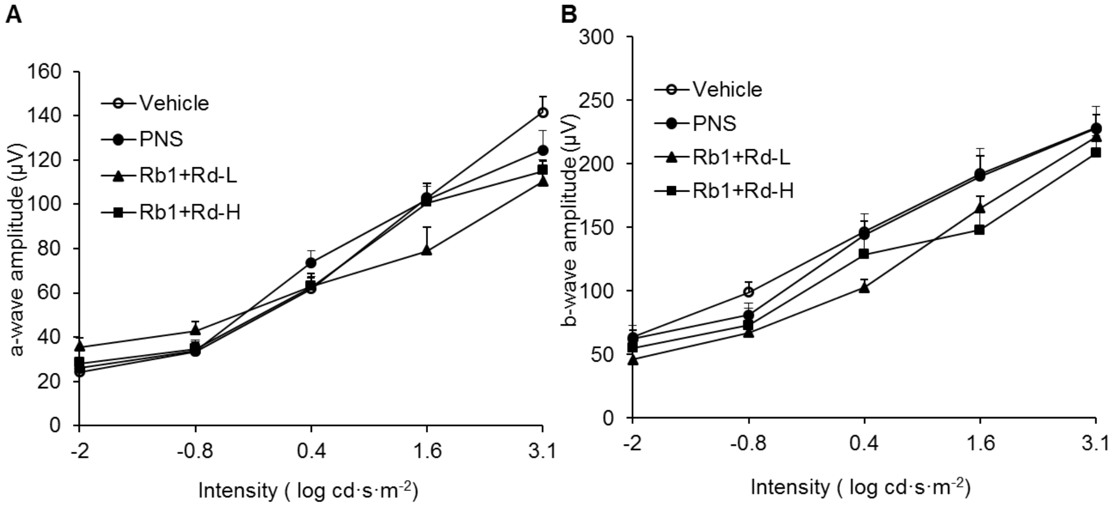

Supplementary Fig. S4

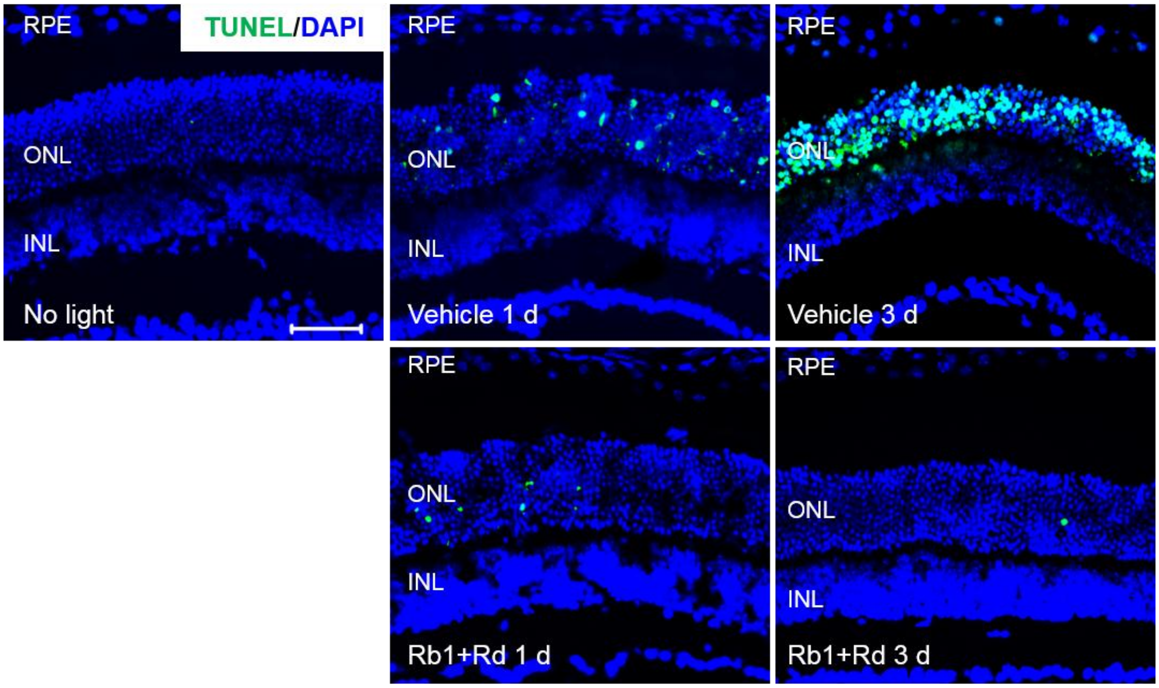

Supplementary Fig. S5

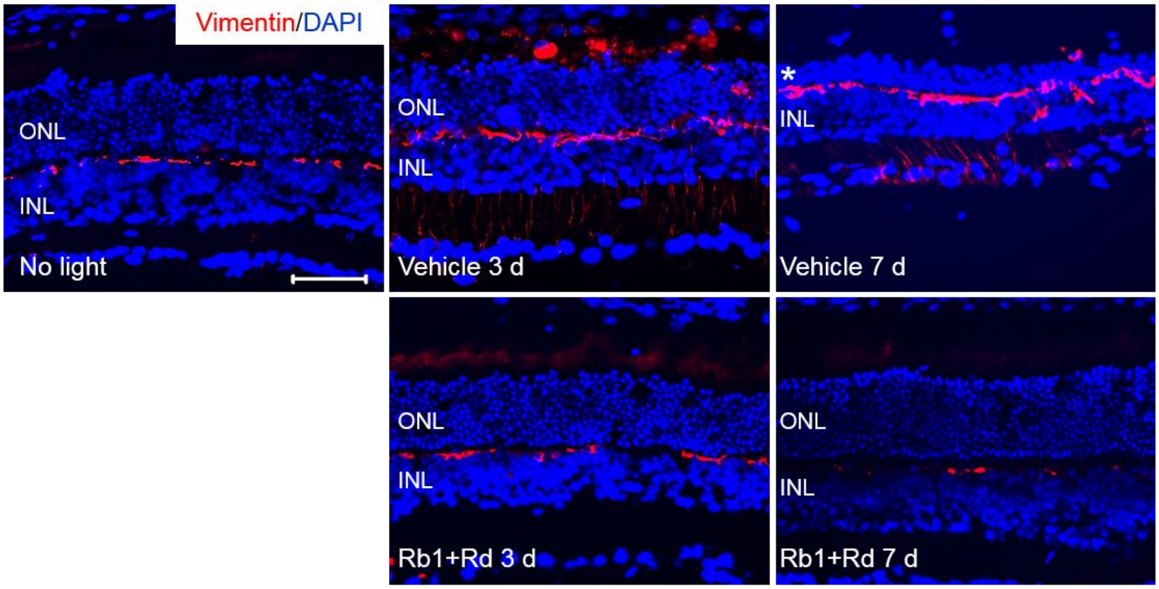

Supplementary Fig. S6

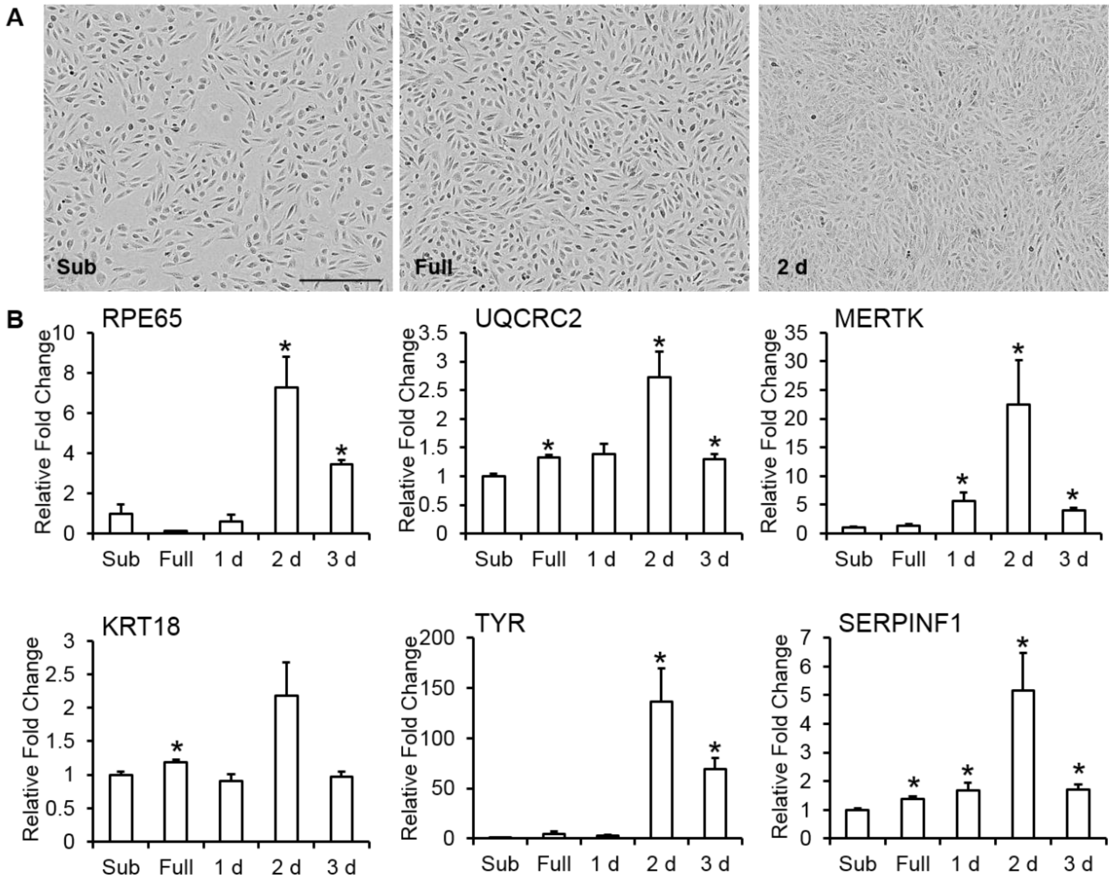

Supplement: Supplementary file 1 — Supplementary Information [file 41598_2017_6471_MOESM1_ESM.pdf]
